# Oncogenic role of miR-155 in anaplastic large cell lymphoma lacking the t(2;5) translocation

**DOI:** 10.1002/path.4539

**Published:** 2015-04-27

**Authors:** Olaf Merkel, Frank Hamacher, Robert Griessl, Lisa Grabner, Ana-Iris Schiefer, Nicole Prutsch, Constance Baer, Gerda Egger, Michaela Schlederer, Peter William Krenn, Tanja Nicole Hartmann, Ingrid Simonitsch-Klupp, Christoph Plass, Philipp Bernhard Staber, Richard Moriggl, Suzanne D Turner, Richard Greil, Lukas Kenner

**Affiliations:** 1 Department of Translational Oncology, National Centre for Tumour Diseases (NCT), German Cancer Research Centre (DKFZ)Heidelberg, Germany; 2 Laboratory for Immunological and Molecular Cancer Research, Third Medical Department, Oncologic Centre, Paracelsus Medical UniversitySalzburg, Austria; 3 Department of Clinical Pathology, Medical University ViennaAustria; 4 Department of Epigenomics and Cancer Risk Factors, German Cancer Research Centre (DKFZ)Heidelberg, Germany; 5 Division of Hematology and Hemostaseology, Comprehensive Cancer Centre Vienna, Medical University of Vienna1090, Vienna, Austria; 6 Division of Molecular Histopathology, Department of Pathology, University of CambridgeUK; 7 Ludwig Boltzmann Institute for Cancer ResearchVienna, Austria; 8 Unit of Pathology of Laboratory Animals, University of Veterinary Medicine ViennaAustria; 9 Institute of Animal Breeding and Genetics, University of Veterinary Medicine Vienna and Medical University of ViennaAustria; 10 European Research Initiative on ALK Related Malignancies (www.erialcl.net)

**Keywords:** miR-155, ALCL, ALK kinase, cytokines, IL-21, IL-22, IL-10

## Abstract

Anaplastic large cell lymphoma (ALCL) is a rare, aggressive, non-Hodgkin's lymphoma that is characterized by CD30 expression and disease onset in young patients. About half of ALCL patients bear the t(2;5)(p23;q35) translocation, which results in the formation of the nucleophosmin-anaplastic lymphoma tyrosine kinase (NPM–ALK) fusion protein (ALCL ALK^+^). However, little is known about the molecular features and tumour drivers in ALK-negative ALCL (ALCL ALK^−^), which is characterized by a worse prognosis. We found that ALCL ALK^−^, in contrast to ALCL ALK^+^, lymphomas display high miR-155 expression. Consistent with this, we observed an inverse correlation between *miR-155* promoter methylation and *miR-155* expression in ALCL. However, no direct effect of the ALK kinase on miR-155 levels was observed. Ago2 immunoprecipitation revealed miR-155 as the most abundant miRNA, and enrichment of target mRNAs *C/EBPβ* and *SOCS1*. To investigate its function, we over-expressed miR-155 in ALCL ALK^+^ cell lines and demonstrated reduced levels of *C/EBPβ* and *SOCS1*. In murine engraftment models of ALCL ALK^−^, we showed that anti-miR-155 mimics are able to reduce tumour growth. This goes hand-in-hand with increased levels of cleaved caspase-3 and high SOCS1 in these tumours, which leads to suppression of STAT3 signalling. Moreover, miR-155 induces IL-22 expression and suppresses the C/EBPβ target IL-8. These data suggest that miR-155 can act as a tumour driver in ALCL ALK^−^ and blocking miR-155 could be therapeutically relevant. Original miRNA array data are to be found in the supplementary material (Table S1). © 2015 The Authors. *The Journal of Pathology* published by John Wiley & Sons Ltd on behalf of Pathological Society of Great Britain and Ireland.

## Introduction

Anaplastic large cell lymphoma (ALCL) is an aggressive non-Hodgkin's T cell lymphoma (NHL) that occurs mostly in children but also in adults. The majority of patients bear the typical t(2;5) aberration, which leads to fusion of the N-terminal oligomerization domain of nucleophosmin (NPM) to the cytoplasmic portion of the anaplastic lymphoma kinase receptor tyrosine kinase (ALK) [1,2]. This results in dimerization and constitutive activation of the kinase and its downstream effectors, including PI3K–AKT [3], JAK3–STAT3 [4,5], MAPK–ERK [6–8], PDGFRB [9] and PLC–PKC [10]. The transforming capacity of nucleophosmin-anaplastic lymphoma kinase (NPM–ALK) has been demonstrated *in vitro*, leading to anchorage-independent growth [11,12], and *in vivo* in diverse engraftment and transgenic mouse models [13–17]. However, not much is known about oncogenic drivers in ALCL without ALK translocations (ALCL ALK^−^), a lymphoma that has a worse prognosis than ALCL ALK^+^ [18]. Despite this relevant difference in clinical outcome, the morphology and gene expression profiles of ALCL are remarkably independent of the presence or absence of the ALK translocation, and only a gene classifier, but no single genes except the ALK kinase, are able to distinguish between the two entities [19–22]. Therefore, the WHO classification published in 2008 provisionally defines ALCL with and without the ALK translocation as two different disease entities, mainly based on the diverging clinical course [23].

However, with better technologies and a deeper examination of the genome, transcriptome and epigenome, some differences between ALCL ALK+ and ALK^−^ have begun to emerge. At the genomic level, deep sequencing identified the t(6;7)(p25.3;q32.3) translocation in 18% of ALCL ALK^−^ patients [24]. More significantly, single-nucleotide polymorphism (SNP) profiling of primary ALCL tissues has revealed strikingly higher levels of genomic instability in ALCL ALK^−^ as compared to ALCL ALK^+^. This was reflected in *p53* loss as a result of the 17p13.3-p12 lesion in 42% of ALCL ALK^−^ compared to only 9% of ALCL ALK^+^ patients, and in keeping with the negative regulation of p53 by NPM–ALK [25]. The second most common deletion was 6q21 (56% versus 6% in ALCL ALK^−^ versus ALK^+^, respectively), resulting in deletion of the B cell differentiation factor BLIMP1, which is known to be disrupted in many cases of activated B cells, such as diffuse large B cell lymphoma [26]. Analysis of the transcriptome has also been informative, in particular a recent study comprising 372 peripheral T cell lymphoma (PTCL) patients, including 31 ALCL ALK^+^ and 32 ALCL ALK^−^ patient samples, that identified 29 genes that differentiated ALCL ALK^+^ from ALCL ALK^−^, although the overall molecular profile was similar between the two ALCL sub-entities [27]. At the level of non-coding RNAs, the miR-17-92 cluster is more highly expressed in ALCL ALK^+^, whereas miR-155 is elevated in ALCL ALK^−^ [28]. The latter has been corroborated by a recent study that used RNA-ISH to detect miR-155 in ALCL specimens and, in addition, found colocalization with neoplastic lymphoma cells [29]. Moreover, ALK regulation of the miR-17-92 cluster, and its ability to partially rescue STAT3 knockdown in ALCL engraftment models, has been reported [30].

The function of miR-155 in ALCL ALK^−^ and other mature T cell lymphomas remains unexplored, but it is known that miR-155 is essential for T cell differentiation and immunity. Moreover, microRNA-155 was the first microRNA (miRNA) to be shown to cause lymphoma in mouse models in two independent studies [31,32].

In this paper, we propose miR-155 as a tumour driver in the majority of ALCL ALK^−^ cases and demonstrate its functions in ALCL cell lines. We show active regulation of interleukin production by miR-155 and that inhibition of miR-155 leads to reduced growth of ALCL ALK^−^ tumours in murine engraftment models.

## Materials and methods

### Cell lines and primary tumour tissues

Formalin-fixed, paraffin-embedded (FFPE) tumours were kindly provided by the Institute of Clinical Pathology at the Medical University of Vienna, after receipt of informed patient consent and in accordance with the Declaration of Helsinki. miRNAs were isolated from 3–5 µm-thick sections, using the RNeasy Mini Kit (Qiagen) according to the manufacturer's instructions. RNA from FFPE lymph nodes from nine healthy age-matched controls was used as reference material. ALCL cell lines containing the ALK translocation SR786 (DSMZ No. ACC 369), SU-DHL-1 (DSMZ No. ACC 356), SUP-M2 (DSMZ No. ACC 509) and Karpas-299 (DSMZ No. ACC 31), as well as the T cell lymphoma cell line JURKAT (DSMZ No. ACC 282), were obtained from the German Centre for Strains and Media (DSMZ, Braunschweig, Germany). Cell lines were gratefully obtained from Marshall Kadin, USA (Mac1 and Mac2a) and Annarosa del Mistro, Padua, Italy (FE-PD). Cell proliferation was measured using the cell titre glow assay (Promega), according to the manufacturer's instructions. Chemiluminescence was assessed using the Infiniti 200 Tecan Reader (Salzburg, Austria).

### Western blotting

For western blots, cell pellets frozen at −80 °C were lysed in RIPA buffer and 30 µg protein/lane was subjected to sodium dodecyl sulphate–polyacrylamide gel electrophoresis (SDS–PAGE). Proteins were transferred to a PVDF membrane and probed with primary antibodies against C/EBPβ (LAP) no. 3087, SHIP1 (C15C9) no. 2725, PTEN no. 9552, Cyclophilin A no. 2175, PTEN no. 9552 and SOCS1 (A156) no. 3950 (all from Cell Signaling Technologies, Danvers, MA, USA), followed by the appropriate horseradish peroxidase-conjugated anti-mouse or anti-rabbit secondary antibodies (Amersham Life Science) for 1 h. Enhanced chemiluminescence detection was performed according to the manufacturer's instructions (Amersham Life Science).

### Ago2 immunoprecipitation

Ago2 immunoprecipitation (Ago2 IP) was performed in three biological replicates, as described [33], with minor modifications as outlined in Supplementary materials and methods (see supplementary material). Overall, three fractions of RNA were isolated from IgG1 IP (IgG) and Ago2 IP (Ago2), as well as total lysate (TL), for further analysis. 300 ng each of *TL*, *IgG* and *Ago2* RNA was reverse-transcribed and quantified using the TaqMan Array system v. 3 (Applied Biosystems), according to the manufacturer's protocol. A list of detected miRNAs, including *C*t values of the three individual experiments and their relative enrichment in the precipitation process, is given in Table S1 (see supplementary material).

### miRNA quantification

Quantitative reverse transcription polymerase chain reaction (qRT–PCR) of miRNAs was performed using RNA isolated by miRNeasy or miRNeasy FFPE kits (Qiagen). For experiments in which individual miRNAs were monitored, relative amounts of miRNA were calculated in relation to miR-92, which was stable in all samples analysed.

### Promoter methylation analysis

Briefly, 500 ng–1 µg genomic DNA was converted using sodium bisulphite and the EZ DNA Methylation kit (Zymo Research). Subsequently, regions of interest were amplified by PCR (for primer sequences, see supplementary material, Table S2), *in vitro* transcribed and base-specifically cleaved by RNase A. Cleavage products were subjected to MALDI–TOF mass spectrometric analysis. Methylation standards were included to ensure a full dynamic range of measurements. The analysed regions (14, 15, 16, 18; primers in Table S2) were chosen to cover the miR-155 promoter CpG island and the adjacent regulatory regions [34].

### MicroRNA transfection

Cells (2.5 × 10^5^) were transfected with miRNA mimics (pre-miR-155, PM12601, anti-miR-155, AM12601; Ambion) or negative control oligonucleotides using Lipofectamine 2000 (Invitrogen), according to the manufacturer's instructions. The final concentrations of anti-miR-155 and the miRNA mimic in the transfection mix were 250 and 50 nm, respectively. Transfection efficiency was validated by qPCR.

### ELISA

For ELISA, the ELISA Ready-Set-Go! Coat-It-Yourself kit (eBioscience) was used according to the manufacturer's manual. In short, wells of a 96-well plate were coated with capture antibody (IL-22, clone 22 URTI; or IL-8, clone 8 CH), washed, blocked and incubated with the samples and standards (eBioscience). After a subsequent wash step, the wells were incubated with the detection antibody, which is conjugated to biotin; the HRP detection enzyme binds to biotin via its conjugated avidin. After addition of the substrate solution, the plate was measured with a 680XR ELISA reader (BioRad) in the linear phase at 450 nm.

### Murine xenografts

A murine xenograft model was established by injecting 5 × 10^6^ Mac1 or Mac2a cells into the right flank of non-obese diabetic (NOD) severe combined immunodeficient (SCID) mice, aged 6 weeks [35]. Injected cell lines had been treated with antisense miRNA mimics, control RNA, pre-miRNA mimics for miR-155 (Ambion) and cultured for 3 days to achieve complete knockdown. miR-155 re-introduction or down-regulation was verified on the day of injection by qRT–PCR. Visible tumours developed at days 21 and 40 for the Mac1 and Mac2a cell lines, respectively, then the mice were killed, the tumours excised and the sizes and weights of the tumours determined. Xenograft studies were performed with approval of the local ethics committee.

### Immunohistochemical staining

Tissue samples were fixed in 4% paraformaldehyde, embedded in paraffin wax and 2 µm sections were prepared. The sections were dewaxed and a steamer pretreatment in Tris–EDTA buffer (Dako) was performed. Endogenous peroxidase activity was quenched by incubation in 3% hydrogen peroxide in phosphate-buffered saline (PBS). For blocking steps, avidin (Sigma-Aldrich), biotin (Sigma-Aldrich) in PBS, and a super block (IDlabs Biotechnology) were used. PY-STAT3 (Cell Signaling, no. 9145), C/EBPβ (sc-150) and SOCS1 (sc-9021; both Santa Cruz Biotechnology, Santa Cruz, CA, USA) were incubated at 4 °C overnight. Immunohistochemical (IHC) detection was performed using the IDetect Super Stain System HRP (IDlabs Biotechnology). Specific signals were amplified using 3-amino-9-ethylcarbazole (IDlabs Biotechnology) under visual control, followed by counterstaining with haematoxylin. Quantitative analysis of the staining was performed using HistoQuest™ software (TissueGnostics GmbH; http://www.tissuegnostics.com; Austria) [36].

### Statistical analysis

For group comparison, an unpaired, two-sided Student's *t*-test was used. Unless indicated otherwise, experiments were performed at least three times and the standard deviation (SD) of means is depicted in the graphs. mRNA expression values were calculated using the 2^–*ΔΔC*t)^ method. Depicted are expression values normalized OD at 260 nm. *GAPDH* and miR-92 were used as standards for mRNAs and miRNAs, respectively. For Ago2 IP experiments, the numbers given in Figure 2 represent the mean of three independent experiments. *C*t values for individual experiments are given in Table S1 (see supplementary material).

## Results

### miR-155 is over-expressed in ALCL patient tumours and ALCL cell lines lacking ALK translocations

**Figure 1:**
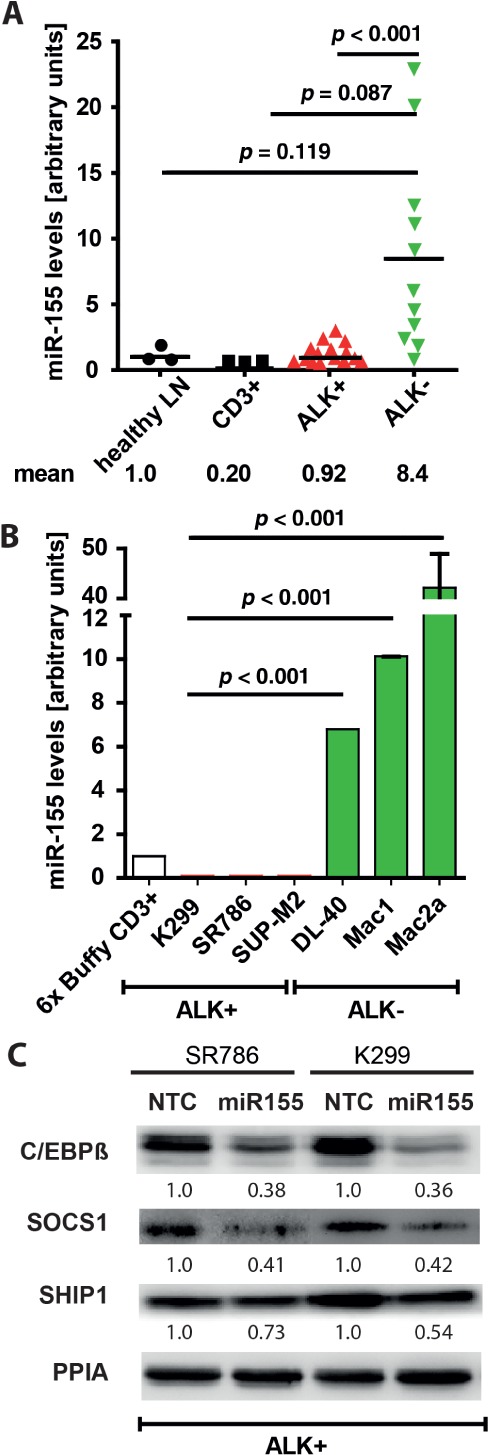
Expression of miR-155 in ALCL tissue and regulation of its target proteins C/EBPβ and SHIP1. (A) miR-155 expression in primary FFPE samples from ALCL patients with (*n =* 15) and without (*n =* 11) the ALK translocation (normalized to healthy human lymph nodes) as well as in (B) six ALCL cell lines with and without ALK translocation. miR-92 was used for standardization [28]; error bars represent mean ± SD. (C) miR-155 mimics were transfected into ALK-positive ALCL cell lines SR786 and Karpas-299: 3 days after transfection, cells were lysed, subjected to gel electrophoresis and probed by western blot with antibodies against the miR-155 targets C/EBPβ, SOCS1 and SHIP1; a representative western blot of three independent experiments is shown

Employing an independent cohort of ALCL patient tumours, we show that miR-155 expression is significantly higher in ALK^−^ (*n =* 11) as compared to ALCL ALK^+^ tumours (*n =* 15, *p* < 0.001) or normal lymph node and CD3^+^ T cells, corroborating our previous results (Figure 1A) [28]. These data are consistent with those from ALCL cell lines, whereby miR-155 expression was assessed in three ALK^−^ and three ALK^+^ ALCL cell lines and CD3-purified primary human T cells; miR-155 was expressed 7-, 10- and 42-fold higher in ALK-negative DL-40, Mac1 and Mac2a ALCL cell lines, respectively, as compared to normal CD3 purified T cells (Figure 1B). In contrast, the ALCL ALK^+^ cell lines Karpas-299, SR786 and SUP-M2 expressed miR-155 at < 5% of the level observed in CD3-purified T cells. These data are in keeping with the primary patient tumour data, whereby miR-155 expression is more prevalent in ALCL ALK^−^ cases. We next determined whether transfection of ALCL cell lines expressing low basal miR-155 (SR786, Karpas-299) with pre-miR-155 is able to actively alter the level of its target proteins. Following the re-introduction of miR-155 into these cell lines, we observed a reduction in expression levels of the miR-155 targets C/EBPβ to 38% and 36%, and SOCS1 to 41% and 42%, of control levels. For SHIP1 the effect was less clear (Figure 1C). Next, we examined expression levels of the miR-155 targets C/EBPβ, SOCS1, SHIP1 and FOXO3a in ALCL cell lines (see supplementary material, Figure S1A). C/EBPβ, but none of the other proteins, was low in the ALCL ALK^−^ Mac1 and Mac2a and high in the ALCL ALK^+^ K299 and SR786 cell lines.

### miR-155 and mRNAs coding for CEBPβ and SOCS1 are part of the Ago2 complex

**Figure 2:**
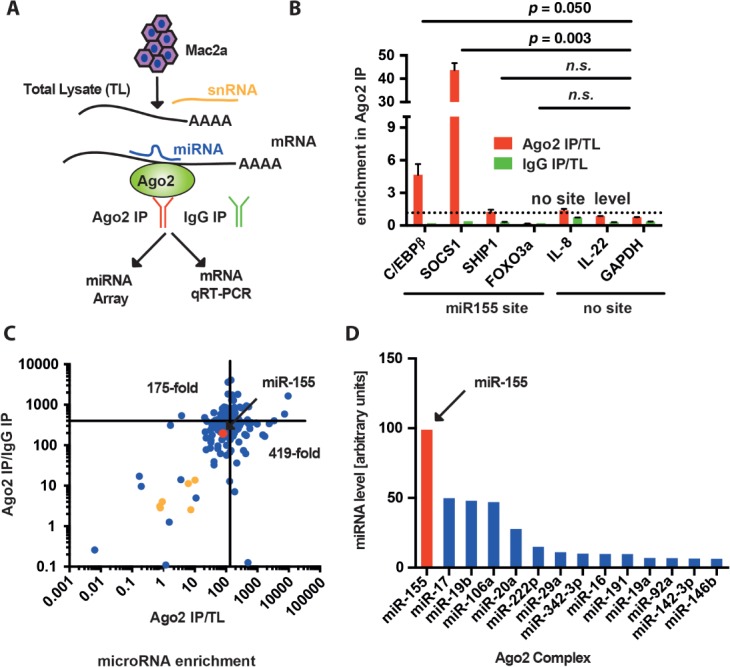
miR-155 in the Ago2 complex. (A) Schematic of the Ago2 IP experiment: immunoprecipitation of miRNAs binding to the Ago2 protein was performed in the Mac2a cell line and RNA from the total lysate (TL), the IgG control (IgG) and the Ago2 immunoprecipitation (Ago2) was isolated. (B) Enrichment of mRNAs with miR-155 binding sites in the 3′-UTR (*C/EBPβ*, *SOCS1*, *SHIP1* and *FOXO3a*) in the Ago2 complex compared to mRNAs without (*IL-8*, *IL-22* and *GAPDH*); the mean level of the latter (dotted line) has been set to 1 for standardization. (C) Relative enrichment of microRNAs in the Ago2 IP complex: each dot represents a microRNA (blue) and its relative enrichment compared to the IgG control (*y* axis) or TL (*x* axis); red, miR-155; yellow, small nucleolar RNA. (D) miR-155 (red bar) is the most abundant microRNA in the Ago2 complex, as measured by Taq-Man qRT–PCR

To elucidate which microRNAs in the context of ALCL are bound to Ago2, and are thus functional, we performed Ago2 immunoprecipitation (IP) from the Mac2a cell line. RNA was isolated from the total lysate (TL), the IgG control IP (IgG) and the Ago2 IP fraction, and global miRNA profiling was performed using Taq Man Array qRT–PCR (Figure 2A). In addition, we probed for mRNA levels of genes with miR-155 binding sites in their 3′-UTRs (*C/EBPβ*, *SOCS1*, *SHIP1* and *FOXO3a*), as well as controls without (*IL-8*, *IL-22* and *GAPDH*) in the Ago2 IP complex. We found significantly enriched *SOCS1* and *C/EBPβ* mRNA in the Ago2 IP, whereas *Foxo3a* and *SHIP1* were not enriched, similar to the controls (Figure 2B). Therefore, we concluded that, in the context of ALCL, C/EBPβ and SOCS1 are the main targets of miR-155, consistent with the data presented in Figure 1C. As expected, we found a strong mean enrichment of miRNAs in the Ago2 complex, which was 419-fold compared to IgG IP and 175-fold compared to TL (Figure 2C). This is also reflected in the numbers of miRNAs above the detection limit in Ago2, IgG and TL fractions, which were 318, 262 and 253, respectively (see supplementary material, Figure S1B). The control small nucleolar RNAs (*RNU44*, *RNU48*, *U6* snRNAs; yellow dots), but also several miRNAs, were not strongly enriched (<10-fold, Figure 2C; see also supplementary material, Table S1). Importantly, miR-155 was enriched 79-fold (compared to total lysate; red dot, Figure 2C) and the most abundant microRNA in the Ago2 complex, suggesting active regulation of target proteins (Figure 2D; see also supplementary material, Table S1).

### miR-155 expression is inhibited by ALK-independent promoter methylation in ALCL ALK^+^ cell lines

**Figure 3:**
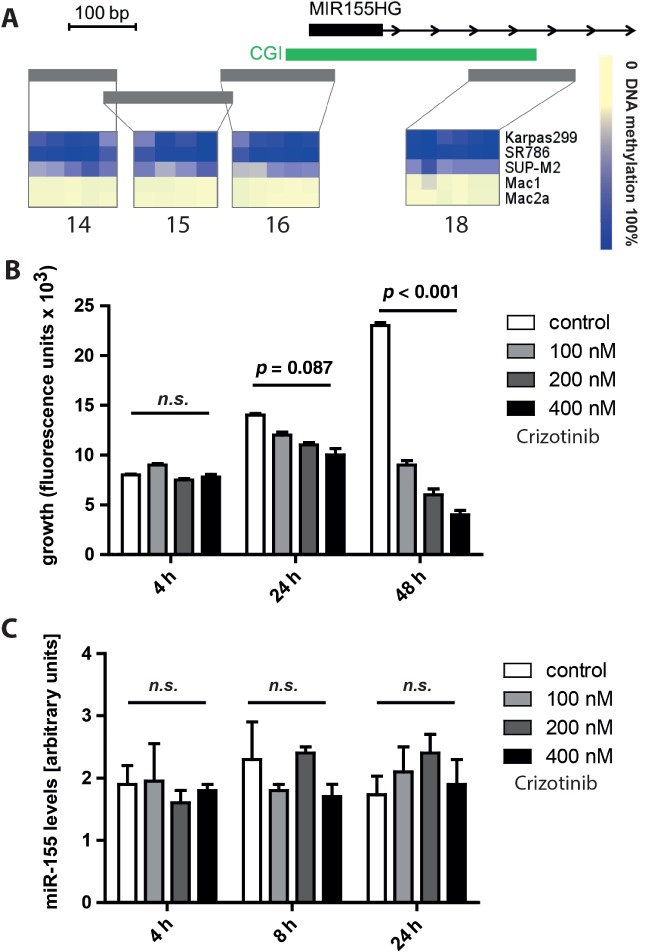
Regulation of miR-155. (A) Using the mass-array technique, we measured promoter DNA methylation in the miR-155 host gene promoter (MIR155HG) CpG island (16) and adjacent regulatory regions (14, 15, 18); data represent the mean of two biological replicates. (B) The ALCL ALK+ cell line Karpas-299 was treated with the indicated concentrations of crizotinib and proliferation, represented by intracellular ATP levels, was measured using a cell titre glow assay at the indicated time points. (C) Using qRT–PCR, miR-155 levels were assessed 4, 8 and 24 h after crizotinib treatment. For (B, C), data represent the mean of three biological replicates. Error bars represent mean ± SD

The methylation status of three regions upstream of the miR-155 host gene start site (14, 15, 16) correlates with miR-155 expression levels [34]. Using bisulphite conversion, RNAse cleavage and MALDI–TOF mass spectroscopic analysis, we analysed the methylation status of these regions in two ALCL ALK^−^ cell lines with high miR-155 expression levels (Mac1, Mac2a) and three ALCL ALK^+^ cell lines with low miR-155 expression levels (Karpas-299, SR786, SUP-M2). In the Mac1 and Mac2a cell lines, low levels of promoter methylation were present (0–5%), whereas the Karpas-299, SR786 and SUP-M2 promoters were highly methylated (70–100%) at all four regions analysed, suggesting that promoter methylation contributes to miR-155 regulation in ALCL (Figure 3A).

To test for an active suppressor function for ALK on miR-155, we inhibited ALK with the small molecule inhibitor crizotinib in the ALK^+^ Karpas-299 cell line and measured growth and miR-155 expression at three different time points (4, 8 and 24 h). Due to the delayed effect of ALK inhibition on miR-155 promoter methylation, we also included a 120 h time point (see supplementary material, Figure S2). Despite the expected growth-reducing effect of crizotinib, no significant increase in miR-155 was observed, suggesting no direct link between ALK kinase activity and miR-155 expression (Figure 3B, C).

### Antagonists of miR-155 reduce tumour growth in murine xenograft models of ALCL ALK^−^

**Figure 4:**
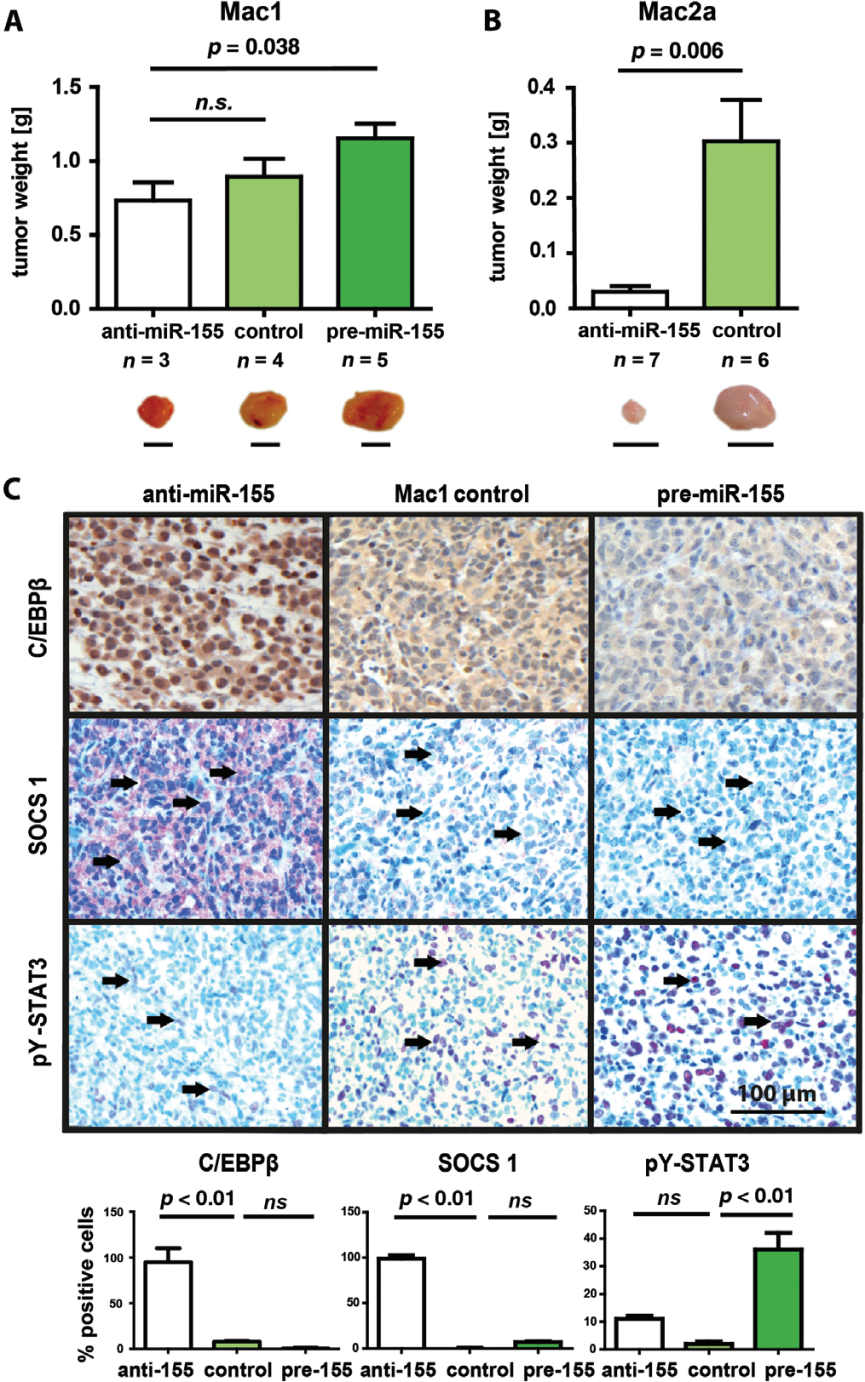
Abrogation of miR-155 reduces tumour growth in an ALCL ALK^−^ mouse model. The ALCL ALK^−^ cell lines Mac1 (A) and Mac2a (B) were transfected with either with anti-miR-155, control-RNA or pre-miR-155 oligonucleotides. Effective knock-down and knock-in was verified by qRT–PCR (see supplementary material, Figure S3). Cells (5 × 10^5^) were injected subcutaneously under the right flank of NOD/SCID mice; the mice were sacrificed after 21 and 40 days for experiments with Mac1 and Mac2a cell lines, respectively, the tumours excised and tumour sizes and weights determined; error bars represent mean ± SD. (C) C/EBPβ, SOCS1, pY-STAT3 immunohistochemistry IHC in murine tumours from Mac1 engraftment experiments containing different initial miR-155 levels: tumours were analysed by IHC (*n =* 4); magnification = ×20; scale bar = 100 µm; arrows, SOCS1 or pY-STAT3 expression in tumour cells. C/EBPβ, SOCS1 and pY-Stat3 IHC expression levels were quantified using HistoQuestTM software (TissueGnostics) [36]

To study the role of miR-155 in ALCL without ALK translocation, we used the cutaneous ALCL ALK^−^ cell line Mac1, which was transfected with either an antisense miRNA-155 mimic, control RNA or a pre-miRNA-155 mimic. Efficiency of knock-out and knock-in was confirmed 2 days later by qRT–PCR (see supplementary material, Figure S3A). Cells (5 × 10^6^ Mac1 cells) were injected subcutaneously under the right flank of NOD–SCID mice and, after 21 days, the mice were sacrificed, the tumours excised and tumour weights determined. Mice that received cells treated with anti-miR-155 had the smallest detectable tumours, whereas mice that received pre-miR-155 treated cells had much larger tumours (*p* = 0.038, Figure 4A). Mice that received control RNA-transfected cells had intermediate tumour sizes, which indicates that miR-155 provides a tumour growth-promoting function. To corroborate these findings, another ALCL ALK^−^ cell line, Mac2a, was employed. The experiments were performed as described above. In this case, the mice transplanted with anti-miR-155-transfected cells developed tumours that were only 10% of the size of tumours in the control group, thus supporting the potent anti-tumour effect of anti-miR-155 in this ALCL model system (*p* = 0.006; Figure 4B). To study more closely the mechanism by which anti-miR-155 mimetics reduce tumour growth, we assessed expression of miR-155 and its targets, C/EBPβ and SOCS1, in the murine tumours. Despite no difference in miR-155 expression (see supplementary material, Figure S3B), immunohistochemical analysis of C/EBPβ and SOCS1 showed significantly higher expression in tumours obtained from mice grafted with the anti-miR155-transfected Mac1 cells (Figure 4C; *p* < 0.01). Notably, the tumours obtained from cells transfected with anti-miR-155 displayed higher levels of cleaved caspase 3, suggestive of miR-155-induced cell survival signals, or at least inhibition of apoptosis (see supplementary material, Figure S4). Since high SOCS1 levels have been described to suppress pY-STAT3, we looked at the activity of STAT3 in these tumours. Indeed, pY-STAT3 levels were highest in the pre-miR-155-transfected group and low in the control group, suggesting an active miR-155–SOCS1–pYSTAT3 axis in these tumours (Figure 4C).

### IL-8 and IL-22, but not IL-10 and IL-21, are expressed in a miR-155-dependent manner in ALCL

**Figure 5:**
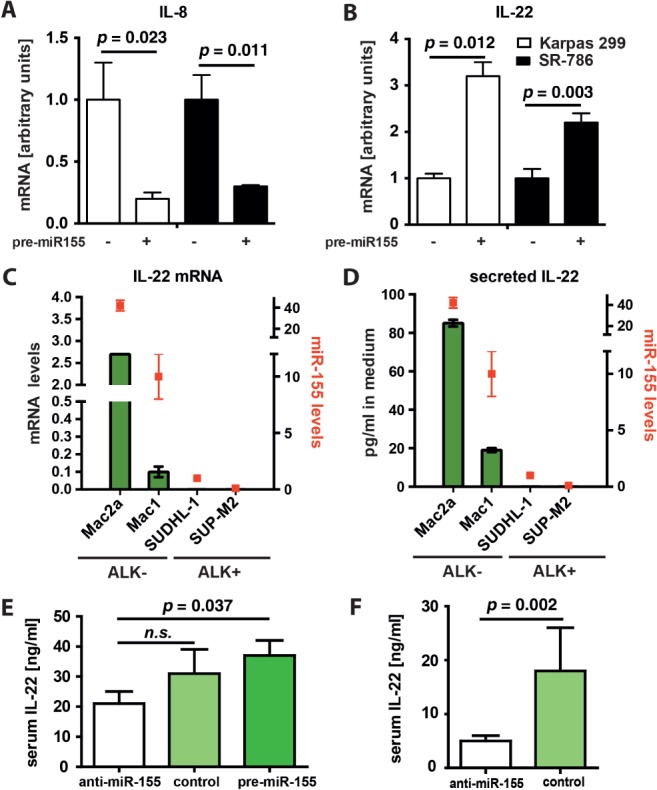
miR-155 influences cytokine expression in ALCL. ALCL ALK^+^ cell lines Karpas-299 and SR786 were transfected with pre-miR155-mimics (+) or non-targeting control RNA (−). After 3 days, RNA was isolated, reverse-transcribed and IL-8 (A) and IL-22 (B) transcript levels were measured; data represent mean and SD of three replicates and are representative of two experimental repeats. (C) RNA was isolated and reverse-transcribed from four ALCL cell lines, and IL-22 and miR-155 levels were determined by qRT–PCR. (D) Basal levels of IL-22 protein were measured by ELISA with growth media of four ALCL cell lines. (E, F) Blood samples were collected at the time of tumour excision from the mice described in Figure 3; serum IL-22 levels were determined from mice engrafted with (E) Mac1 and (F) Mac2a cell lines, by ELISA; error bars represent mean ± SD

Because of the described role of miR-155 in the immune system, we assumed that miR-155 might modulate the tumour environment via cytokines [37]. Therefore, we assessed the effect of miR-155 over-expression on the expression and secretion of a cytokine subset (IL-8, IL-10, IL-21 and IL-22) described in ALCL [20,38,39]. When Karpas-299 and SR786 cells were transduced with pre-miR-155, IL-8 transcript levels were reduced by 80% in both cell lines (Figure 5A). Similarly, IL-8 protein levels in the growth medium were reduced after miR-155 transfection in the Karpas-299 cell line (see supplementary material, Figure S5A). In the SR786 cell line, IL-8 protein levels were below the detection limit (data not shown). Consistently, basal levels of IL-8 were low in ALCL ALK^−^ cell lines (which have high miR-155 expression levels), as compared to ALCL ALK^+^ cell lines (with low miR-155 expression; see supplementary material, Figure S5B).

In contrast, IL-22 transcript levels were induced 3.2- and 2.2-fold, respectively, following pre-miR155 transfection (Figure 5B), whereas levels of IL-10 and IL-21 were not significantly affected (see supplementary material, Figure S5C). To determine steady-state levels, we monitored *IL-22* mRNA in a panel of ALCL cell lines and correlated it to miR-155 levels (Figure 5C). In parallel, we analysed IL-22 protein levels in the growth medium by ELISA and detected secreted IL-22 protein in the range 15–85 ng/ml (Figure 5D). The IL-22 receptor under non-neoplastic conditions is exclusively expressed in non-haematopoietic cells. However, when we studied its expression in ALCL cell lines, we found expression of IL-22R in five of seven cell lines, suggesting active IL-22 signalling in these tumours (see supplementary material, Figure S5D).

In addition, IL-22 levels in the serum of mice grafted with Mac1 cells transfected with anti-miR155 were significantly lower compared to mice grafted with the pre-miR155 transfected cells (*p =* 0.037, Figure 5E). Consistent with this finding, mice carrying the Mac2a cells transfected with anti-miR-155 also had significantly lower IL-22 levels than the control mice (*p =* 0.002, Figure 5F). These data suggest that IL-22 is a miR-155-modulated tumour growth-associated cytokine in ALCL.

### miR-155 targets in primary ALCL tissue

**Figure 6:**
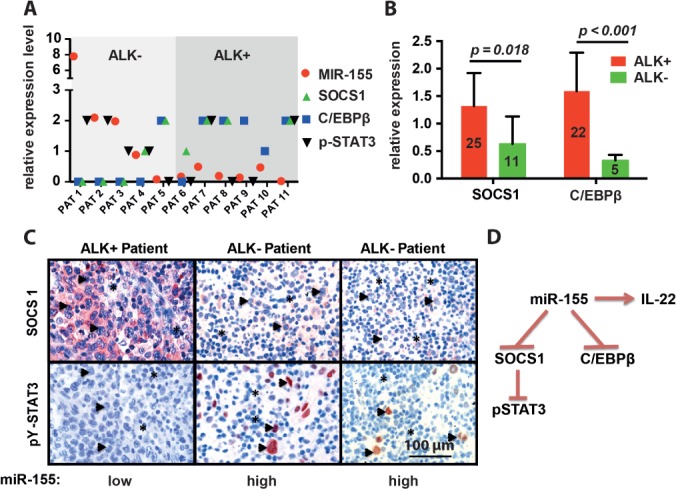
miR-155 targets SOCS1 and C/EBPβ in primary human tumour material. (A) C/EBPβ, SOCS1 and pY-STAT3 were assessed by IHC and miR-155 by qRT–PCR in 11 human ALCL primary tissue samples; PAT, patient. (B) SOCS1 and C/EBPβ IHC levels were assessed in 36 and 27 FFPE ALCL tissue specimens, respectively, with (red) and without (green) the ALK translocation. (C) Representative IHC pictures of patient 8 (ALK^+^), patient 1 (ALK^−^) and patient 2 (ALK^−^) are shown; magnification = ×20. (D) Schematic drawing depicting our current view of miR-155 as an inducer of IL-22 and, at the same time, a negative regulator of C/EBPβ and SOCS1. SOCS1 suppression may lead to pY-STAT3 activation in a significant subset of ALCL ALK^−^ patients

To investigate the relevance of our data, we determined miR-155, C/EBPβ, SOCS1 and pY-STAT3 in 11 human ALCL primary patient tissue samples. We showed high miR-155 expression in four of five ALK^−^ and low miR-155 expression levels in six of six ALK^+^ samples (Figure 6A). In all cases analysed, we could see an inverse correlation of miR-155 with C/EBPβ and SOCS1 (Figure 6A, B). Interestingly, patient 5, who had unusually low miR-155 expression for an ALCL ALK^−^ case, displayed enhanced SOCS1 and C/EBPβ expression, in line with our observed regulation of these genes by miR-155. In four of five ALCL ALK^−^ cases, we could see an inverse correlation of SOCS1 to pY-STAT3, which suggests that miR-155 suppresses STAT3 activation via SOCS1 in a significant subgroup of ALCL ALK^−^ cases. We also assessed SOCS1 and C/EBPβ in a significant number of primary FFPE ALCL samples. Both proteins were expressed at significantly lower levels in ALCL ALK^−^ compared to ALCL ALK^+^ patient samples. Figure 6C depicts representative IHC images from patients 8, 1 and 2. In summary (Figure 6D), we propose that in ALCL ALK^−^ cells miR-155 suppresses its direct targets SOCS1 and C/EBPβ and induces IL-22. This leads to pY-STAT3 activation in a significant sub-fraction of the patients.

## Discussion

We have unravelled the functional consequences of the differential miRNA expression observed in ALCL ALK^−^ versus ALCL ALK^+^ tumour cells. In particular, miR-155 is expressed nine-fold higher in ALCL ALK^−^ as compared to ALCL ALK^+^ cells. Analysing potential miR-155 target proteins in ALCL, we found that C/EBPβ and SOCS1 are suppressed upon miR-155 re-introduction in ALCL ALK^+^ cell lines. C/EBPβ levels correlated inversely with miR-155 expression in ALCL cell lines. In primary patient tissue it has been shown at both mRNA and protein levels that C/EBPβ is expressed at significantly lower levels in ALCL ALK^−^ as compared to ALCL ALK^+^, which corroborates our data [20]. We extended these findings by also showing suppression of the miR-155 target SOCS1 in ALCL ALK^−^.

When we analysed interleukins IL-8, IL-10, IL-21 and IL-22, which have been reported to play a role in autocrine or paracrine stimulation of ALCL, we found that IL-8 is reduced and IL-22 is induced by miR-155 over-expression; IL-10 and IL-21 levels remained unchanged. The functional consequences of miR-155 depletion were demonstrated in ALCL mouse models that highly expressed miR-155, but not NPM–ALK, in their tumours. Mac1 or Mac2a ALCL ALK^−^ cell lines were engrafted into immune-deficient NOD–SCID mice. Before engraftment, the cells were treated with an anti-miR-155 mimic, control RNA or pre-miR-155 mimic. Interestingly, tumour growth was directly related to miR-155 levels in the injected cells. At the time point when tumours were harvested, miR-155 levels could not be differentiated between the groups, indicating dilution of miRNA mimics during tumour growth. In contrast, when we analysed the tumours of the mice, we found high levels of the miR-155 targets SOCS1 and C/EBPβ and low pY-STAT3 levels in anti-miR-155-treated tumours, suggesting long-term target regulation. In order to elucidate how miR-155 is regulated in ALCL, we analysed DNA methylation of the promoter of the miR-155 host gene. In all five ALCL cell lines analysed, promoter methylation was inversely correlated to expression of miR-155. The greater genomic instability that has been demonstrated for ALCL ALK^−^ with high-density SNP arrays is remarkable, and the most frequent deletion found was 6q21 in 56% of ALCL ALK^−^ cases, encompassing the *PRDM1* gene coding for BLIMP1. Accordingly, BLMP1 over-expression led to reduced growth and apoptosis induction in an ALCL ALK^+^ cell line. When BLIMP1-regulated genes were analysed, it was found that miR-155 and interferon regulatory factor 4 (IRF-4) are repressed by BLMP1 [26]. So, in a del6q21 situation as found in 58% of ALCL ALK^−^ cases, BLMP1 loss may contribute to high miR-155 expression in ALCL ALK–.

In ALCL ALK^+^, IL-22 was able to stimulate growth, IL22R1 was expressed and IL-22 blocking antibodies reduced growth of the SUDHL1 cell line [38]. This supports the idea of an important function of IL-22/IL-22R1 in ALCL. We extended these studies to ALCL ALK^−^ by showing that high expression of IL-22R1 and IL-22 can also be found in ALCL ALK^−^ cell lines. Accordingly, in this study, we demonstrated that miR-155 is able to induce IL-22 and is expressed at much higher levels in ALCL ALK^−^ primary tissue and cell lines (Figure 1A). A recent study has highlighted a mechanism by which miR-155 induces IL-22. miR-155 represses the DNA binding protein JARID2, which closely interacts with polycomb repressing complex 2 (PRC2) in Th17 cells, thereby leading to derepression of Th17 typical cytokines, such as IL-22 [40]. Indeed, IL-22 is 3.9-fold more highly expressed in ALCL ALK^−^ versus ALK^+^, as revealed by transcriptome analysis of primary tissue [41]. In line with our findings, it was shown that miR-155 knock-out mice have impaired Th17 polarization, resulting in reduced levels of IL-22 secretion after stimulation with anti-CD3 antibody [42,43]. In this pathway there are multiple possibilities for therapeutic intervention, since IL-22R1 heterodimerizes with IL-10R and is known to activate downstream tyrosine kinases TYK2 and JAK1 [44]. TYK2 has recently been shown to be essential in T cell acute lymphoblastic leukaemia (T-ALL) primary cells [45]. A recent publication [46] using integrated genomic sequencing shows gain-of-function mutations in *JAK1*, *JAK3* and *STAT5B* in T cell prolymphocytic leukaemia (T-PLL), supporting the importance of JAK kinases for T cell lymphoma growth. Inhibitors of different JAK kinases and TYK2 are available, but often their specificity is limited. However, these inhibitors are actively being developed further and improved by pharmaceutical companies [47,48].

Deciphering the role of miR-155 in JAK–STAT signalling in ALCL ALK^−^ may be the basis for the introduction of more targeted therapeutics in mature T cell lymphomas, which to date have a dire prognosis.

## Supporting Information

Appendix S1. Supplementary materials and methods

**Table S1.** List of detectable microRNAs in total lysate fraction (TL), IgG control (IgG IP) and Ago2 complex (Ago2 IP). The *C*t values of microRNAs detected in the three fractions of three independent replicates of the TL, IgG IP and Ago2 IP, using the Taq Man Array system v. 3 (Applied Biosystems), are listed. The mean of the three experiments was used to calculate individual miRNA enrichment. Low *C*t value corresponds to high expression of the respective miRNA. Finally, a list of all detectable miRNAs with their fold enrichment in Ago2 IP versus TL, Ago2 IP versus IgG IP and IgG IP versus TL (light yellow) is provided.

**Table S2.** Regions analysed for CpG methylation close to the miR-155 host gene promoter: given are primer pairs of the analysed regions 14, 15, 16 and 18

**Figure S1.** Basal expression of miR-155 targets in ALCL cell lines and number of detectable miRNAs in the Ago2 IP fractions. (A) ALCL, ALK- (FE-PD, Mac1, Mac2a) and ALCL, ALK^+^ (Karpas-299, SR-786, SU-DHL-1) cell lines and the acute myeloid leukaemia cell line Jurkat were lysed, subjected to gel-electrophoresis and probed with antibodies against miR-155 targets SHIP1, C/EBPβ, FOXO3a and SOCS1. Since SHIP1 is a PIP3 lipid-phosphatase similar to PTEN, we also probed for PTEN. Levels of C/EBPβ were inversely correlated with miR-155 expression of cell lines, although the other described targets showed no correlation when basal levels were monitored. (B) After denaturation of proteins in the IP fractions, RNA was extracted and miRNAs were quantified using the Taq Man Array system v. 3 (Applied Biosystems). In the total lysate, IgG and Ago2 fraction 253, 262 and 318 miRNAs were detected, respectively

**Figure S2.** miR-155 expression in response to ALK inhibition. The ALCL, ALK^+^ cell line Karpas-299 was treated for 120 h with 5, 10, 30, 50 and 100 nm crizotinib and cells were counted using the Moxi Z-Mini Automated Cell Counter from ORFLO technologies (Ketchum, ID, USA). In parallel, miR-155 levels were assessed by qRT–PCR. Despite the effective proliferation-reducing effect of crizotinib (*p =* 0.0043), only a small increase in miR-155 levels was observed, which was not significant (*p =* 0.0643); error bars represent mean ± SD

**Figure S3.** miR-155 levels in cell lines used for the murine engraftment experiments. (A) The ALCL cell lines Mac1 and Mac2a were transfected with either anti-miR-155 mimics, pre-miR-155 mimics or non-targeting control RNA: 3 days after transfection, RNA was isolated, reverse-transcribed and probed for expression of miR-155 and miR-92 by qRT–PCR; on the same day, the cells were injected into mice, as described (day 0 of tumour growth). (B) At day 21, the mice were sacrificed and tumours prepared for immunostaining; RNA was isolated, reverse-transcribed and probed for expression of miR-155 and miR-92 by qRT–PCR

**Figure S4.** Cleaved caspase 3 staining in murine tumours: cleaved caspase 3 IHC expression levels were quantified using HistoQuest software (TissueGnostics)

**Figure S5.** miR-155 and cytokine expression in ALCL cell lines. (A) The ALCL cell lines Karpas-299 and SR786 were transfected with pre-miR-155 oligos or control oligos (Ambion): after 3 days, levels of IL-8 in the medium were quantified, using the IL-8 ELISA kit (e-bioscience); the experiment was carried out in five parallel chambers. For the SR786 cell line, IL-8 levels in the medium were below the detection limit. The result for the Karpas-299 cell line is shown. (B) RNA was isolated from different ALCL cell lines, reverse-transcribed and levels of *IL-8* mRNA were determined by qRT–PCR. (C) ALCL, ALK^+^ cell lines Karpas 299 and SR786 were transfected with pre-miR155-mimics (+) or non-targeting control RNA (−): after 3 days, RNA was isolated, reverse-transcribed and *IL-10* and *IL-21* levels were measured by TaqMan qRT–PCR; neither cytokine was influenced by miR-155 transfection. (D) RNA was isolated from different ALCL cell lines, reverse-transcribed and levels of *IL-22R* determined by qRT–PCR; names of cell lines are given
