# Supplementary material for: Oncogenic role of miR-155 in anaplastic large cell lymphoma lacking the t(2;5) translocation
Source: J Pathol. 2015 Apr 27;236(4):445–56. doi: 10.1002/path.4539 (PMC4557053; doi:10.1002/path.4539)
Supplement: Supplementary file 1 — Appendix S1. Supplementary materials and methods [file path0236-0445-sd1.doc]

+A: **Supplementary materials and methods**

+B: Ago2 immunoprecipitation

Briefly, 4 × 10^8^ Mac2a cells were harvested, washed with PBS and lysed in ice-cold polysome lysis buffer (10 mm HEPES, pH 7, 100 mm KCl, 5 mm MgCl_2_, 0.5% NP-40, 1 mm ditihothreitol (DTT), 0.25 U/ml RNAaseOUT, protease inhibitors and 0.4 mm vanadyl ribonucleotide complex) and frozen at –80°C. For antibody loading, 50 µl Protein G Sepharose (GE Healthcare) was washed twice with 500 µl NT2 buffer (50 mm Tris–HCl, pH 7.5, 150 mm NaCl, 1 mm MgCl_2_) and 5 µg Ago2 antibody (Clone2E12-1C9, Abnova) or IgG1 control antibody (Jackson Immunoresearch Antibodies) were added and rotated for 1 h at 4°C in NT2 buffer and finally washed twice with the same buffer. Lysed Mac2a cells were thawed on ice, the supernatant was collected by centrifugation for 15 min at 15 000 × *g* at 4°C and therafter 850 µl NT2 buffer (containing 0.005% NP-40, 0.25 U/ml Rnaseout, 0.4 mm vanadyl ribonucleotide complex, 1 mm DTT, 1 mm EDTA and protease inhibitors) was added. Aliquots for the total lysate (TL) fraction were removed. Lysate was combined with the prepared beads and rotated for 1 h at 4°C. Prior to elution of bound protein, the beads were washed five times with NT2 buffer, prepared as described above, followed by incubation in 50 μl 1 m glycine, pH 2.3, at room temperature for 10 min, followed by neutralization using 5 μl 1 m Tris–HCl, pH 8. The proteins were then degraded by adding 3 μl proteinase K (Qiagen) and RNA was isolated from IgG1 IP (IgG) and Ago2 IP (Ago2) as well as total lysate (TL), using 350 μl Trizol LS reagent (Invitrogen), followed by miRNeasy kit (Qiagen) purification.
