# Supplementary material for: Oncogenic role of miR-155 in anaplastic large cell lymphoma lacking the t(2;5) translocation
Source: J Pathol. 2015 Apr 27;236(4):445–56. doi: 10.1002/path.4539 (PMC4557053; doi:10.1002/path.4539)
Supplement: Supplementary file 2 — Table S1. List of detectable microRNAs in total lysate fraction (TL), IgG control (IgG IP) and Ago2 complex (Ago2 IP). The Ct values of microRNAs detected in the three fractions of three independent replicates of the TL, IgG IP and Ago2 IP, using the Taq Man Array system v. 3 (Applied Biosystems), are listed. The mean of the three experiments was used to calculate individual miRNA enrichment. Low Ct value corresponds to high expression of the respective miRNA. Finally, a list of all detectable miRNAs with their fold enrichment in Ago2 IP versus TL, Ago2 IP versus IgG IP and IgG IP versus TL (light yellow) is provided. [file path0236-0445-sd2.pdf]

	Replicate 1,w1			Replicate 2, w2			Replicate 3, h1		
	TL	IgG IP	Ago2 IP	TL	IgG IP	Ago2 IP	TL	IgG IP	Ago2 IP
**hsa-miR-155-002623**	**21,52**	**25,09**	**15,71**	**21,27**	**26,91**	**16,15**	**21,42**	**15,88**	**13,41**
hsa-miR-19b-000396	22,05	28,15	18,23	21,19	27,99	16,61	22,29	16,50	13,50
hsa-miR-17-002308	22,41	27,58	17,51	21,88	27,43	16,51	22,01	16,33	14,16
hsa-miR-106a-002169	22,59	28,07	17,63	21,78	27,42	16,39	22,43	16,61	14,41
hsa-miR-20a-000580	23,32	29,38	18,83	22,98	29,09	17,41	23,11	17,36	14,41
hsa-miR-142-3p-000464	24,15	32,25	21,92	24,26	31,81	19,99	23,78	17,99	14,44
hsa-miR-525-3p-002385	23,85	28,69	17,21	40,00	40,00	40,00	24,57	19,56	15,13
hsa-miR-222-002276	25,23	29,36	19,22	27,19	33,71	18,74	25,69	19,20	15,19
hsa-miR-29a-002112	25,19	30,33	19,79	23,28	29,26	19,13	24,60	19,37	15,40
hsa-miR-191-002299	25,88	30,48	20,12	25,33	30,14	19,28	25,24	19,94	15,45
hsa-miR-16-000391	25,12	30,31	20,01	24,59	31,23	19,32	25,86	19,26	15,47
hsa-miR-19a-000395	23,89	31,97	21,27	23,43	30,39	19,28	24,27	18,47	15,55
hsa-miR-146b-001097	25,98	31,09	20,59	25,23	30,99	19,85	25,50	20,16	15,97
hsa-miR-342-3p-002260	25,88	29,34	19,81	29,06	34,34	18,43	26,88	20,40	16,45
hsa-miR-21-000397	25,09	31,48	21,07	25,48	32,26	19,67	26,75	20,43	16,48
hsa-miR-24-000402	27,19	29,84	20,39	24,21	29,99	20,37	26,06	20,90	16,57
hsa-miR-92a-000431	18,16	29,40	20,19	25,95	30,05	19,34	27,02	20,36	16,65
hsa-miR-331-000545	27,40	31,90	21,71	26,67	30,89	20,30	27,39	21,33	17,07
U6 snRNA-001973	20,33	23,09	21,07	19,32	23,61	22,55	20,08	18,57	17,11
U6 snRNA-001973	19,65	22,99	20,12	19,31	23,66	22,22	20,15	18,84	17,14
U6 snRNA-001973	19,92	23,07	20,71	19,23	23,75	22,48	20,04	18,44	17,16
U6 snRNA-001973	30,09	23,23	21,10	19,54	23,63	22,76	20,16	18,24	17,19
hsa-miR-320-002277	27,01	29,85	20,44	26,56	30,71	20,22	27,99	21,29	17,37
hsa-miR-484-001821	27,63	36,78	24,05	31,72	21,21	22,77	28,32	21,92	17,52
hsa-miR-30c-000419	20,36	31,14	21,17	26,42	31,37	20,14	27,89	21,53	17,80
hsa-miR-186-002285	28,31	33,09	22,63	28,10	32,11	21,35	28,94	21,91	17,90
hsa-miR-193b-002367	27,29	31,81	22,15	26,95	31,30	21,19	28,35	22,20	18,12
hsa-let-7b-002619	27,21	31,97	21,62	31,03	40,00	23,25	27,16	22,11	18,25
mmu-miR-374-5p-001319	27,17	27,97	22,23	28,43	23,41	21,87	27,97	22,13	18,38
mmu-miR-93-001090	8,36	31,48	21,45	26,71	32,31	20,49	27,51	21,39	18,41
hsa-miR-30b-000602	40,00	34,86	23,08	27,89	31,18	21,51	28,77	22,18	18,42
hsa-miR-106b-000442	27,48	35,34	23,45	27,11	34,04	22,36	28,97	22,35	18,49
hsa-let-7e-002406	27,93	33,81	23,04	27,92	32,83	22,95	27,17	21,99	18,79
hsa-miR-15b-000390	27,88	35,49	23,39	27,51	35,44	22,43	28,59	23,08	18,84
hsa-miR-135b-002261	28,66	33,60	24,16	27,30	32,93	23,04	30,17	23,69	18,88
hsa-miR-708-002341	27,25	31,84	22,42	28,26	35,38	21,64	29,31	22,82	18,88
hsa-miR-425-5p-001516	29,07	32,54	22,99	28,12	32,58	22,19	29,34	23,38	19,00
hsa-miR-18b-002217	29,10	40,00	24,50	30,42	40,00	24,07	29,54	23,53	19,01
hsa-miR-454-002323	28,88	33,20	23,88	28,38	34,06	23,09	28,34	23,20	19,17
hsa-miR-26b-000407	23,34	35,48	24,35	28,47	33,87	23,34	28,96	23,40	19,25
hsa-miR-18a-002422	28,84	33,72	24,36	27,83	33,64	22,73	29,43	22,99	19,26
hsa-let-7g-002282	27,90	34,04	23,44	27,80	35,01	22,97	28,57	23,17	19,37
hsa-miR-28-3p-002446	33,50	32,49	23,36	28,57	32,09	22,42	29,18	23,36	19,59
hsa-miR-590-5p-001984	28,85	34,31	26,04	30,12	35,52	24,20	30,83	24,21	19,59
hsa-miR-29c-000587	40,00	34,26	24,37	28,00	35,03	23,83	29,37	23,95	19,65

hsa-miR-210-000512	27,34	34,41	23,21	26,61	33,55	23,34	28,82	24,36	19,69
hsa-miR-132-000457	27,75	32,74	22,86	28,19	31,44	22,28	29,43	24,35	19,82
hsa-miR-146a-000468	29,88	40,00	24,50	28,97	33,64	22,47	29,66	24,21	19,84
hsa-miR-130b-000456	29,59	40,00	24,38	28,99	40,00	23,87	30,13	23,60	19,90
hsa-miR-26a-000405	29,81	35,12	24,01	29,18	34,39	23,58	30,14	24,08	20,19
hsa-miR-374-000563	30,70	35,78	26,32	30,10	34,53	24,41	30,79	24,71	20,31
hsa-miR-103-000439	30,38	40,00	24,89	29,00	34,38	24,48	30,25	24,64	20,42
hsa-miR-301-000528	29,82	40,00	26,48	30,03	35,06	25,09	30,45	24,51	20,50
hsa-miR-519d-002403	28,58	34,96	22,42	40,00	40,00	40,00	29,27	27,18	20,51
hsa-miR-29b-000413	40,00	40,00	27,91	29,91	40,00	26,42	31,42	24,50	20,56
hsa-let-7a-000377	29,77	35,26	25,18	29,77	40,00	24,54	29,52	23,96	20,58
hsa-miR-660-001515	29,46	40,00	25,45	29,99	30,77	24,60	30,16	24,98	20,71
hsa-miR-197-000497	29,56	32,72	23,32	30,32	34,84	22,45	30,26	24,40	20,74
hsa-miR-27a-000408	40,00	33,89	24,50	29,35	32,65	23,66	31,95	24,63	20,92
hsa-miR-140-3p-002234	33,31	36,96	25,76	33,22	35,49	25,50	33,56	25,23	20,92
hsa-miR-25-000403	29,17	33,56	25,35	30,38	34,31	24,31	30,76	24,75	21,00
hsa-miR-532-001518	32,07	35,35	24,91	31,27	35,57	24,12	31,80	24,85	21,17
hsa-miR-196b-002215	32,18	34,99	26,63	30,48	40,00	25,18	30,39	26,19	21,26
hsa-miR-365-001020	31,68	34,40	25,18	35,03	40,00	27,13	33,62	25,37	21,27
hsa-miR-345-002186	30,92	40,00	25,80	32,50	35,55	24,48	32,46	25,33	21,32
RNU48-001006	24,98	26,09	21,04	23,40	27,49	22,39	26,50	22,58	21,40
mmu-miR-140-001187	29,23	35,59	25,76	28,80	40,00	24,84	30,06	24,38	21,43
hsa-let-7d-002283	30,33	33,04	25,22	30,41	40,00	25,13	30,19	25,36	21,49
hsa-let-7f-000382	29,32	35,96	25,61	29,36	34,10	24,43	30,26	25,19	21,55
hsa-miR-532-3p-002355	31,49	34,47	25,50	30,86	34,98	24,13	32,69	25,81	21,62
hsa-miR-330-000544	30,07	34,34	25,34	31,05	35,02	24,43	31,80	26,31	21,66
hsa-miR-20b-001014	30,11	34,13	25,39	30,94	35,50	24,83	30,69	24,82	21,74
hsa-miR-744-002324	30,13	33,56	25,68	29,77	40,00	26,12	31,00	26,33	21,82
hsa-miR-28-000411	25,69	34,67	26,39	30,13	40,00	25,57	31,30	26,07	22,03
hsa-miR-27b-000409	29,90	40,00	26,04	30,68	40,00	25,23	33,30	25,83	22,05
hsa-miR-486-3p-002093	34,34	40,00	26,87	40,00	40,00	25,60	40,00	26,70	22,19
hsa-miR-34a-000426	40,00	32,14	24,99	27,94	33,24	25,29	29,99	25,75	22,20
hsa-miR-221-000524	34,70	40,00	26,24	31,23	40,00	25,03	31,20	25,94	22,21
hsa-miR-195-000494	30,26	35,48	26,50	31,40	40,00	25,62	32,12	26,32	22,32
hsa-miR-652-002352	31,74	40,00	26,24	31,79	40,00	26,12	33,03	27,01	22,35
hsa-miR-324-3p-002161	31,84	34,56	25,98	30,28	34,72	25,35	32,48	26,33	22,37
hsa-miR-183-002269	31,70	40,00	26,39	33,16	40,00	26,17	32,03	26,32	22,42
hsa-miR-182-002334	31,51	40,00	26,41	32,28	35,94	25,93	31,99	26,69	22,43
hsa-miR-301b-002392	31,21	40,00	27,76	34,23	40,00	28,46	32,26	26,13	22,49
RNU44-001094	24,95	26,98	22,08	24,72	28,68	24,10	27,01	23,66	22,62
hsa-miR-486-001278	30,79	40,00	25,96	31,03	32,33	24,95	32,61	26,57	22,67
hsa-miR-101-002253	34,10	40,00	29,97	33,09	40,00	28,04	33,77	26,63	22,72
hsa-miR-142-5p-002248	32,32	35,82	29,96	34,96	40,00	28,64	31,56	24,87	22,97
hsa-miR-362-001273	35,11	35,41	28,39	32,00	35,04	26,78	35,03	28,42	22,98
hsa-miR-508-001052	35,37	40,00	21,73	40,00	40,00	35,46	40,00	26,14	22,99
hsa-miR-422a-002297	33,74	36,53	27,15	34,10	33,81	29,43	32,87	27,46	23,01
hsa-miR-15a-000389	33,72	40,00	30,81	33,21	40,00	30,55	33,38	28,93	23,35
hsa-miR-98-000577	31,61	34,49	27,33	31,97	40,00	27,14	32,32	27,01	23,37
hsa-miR-23a-000399	34,68	40,00	26,86	31,97	40,00	27,08	33,38	26,28	23,48
hsa-miR-339-5p-002257	32,98	35,17	28,39	33,45	40,00	26,88	40,00	27,74	23,64
hsa-miR-185-002271	32,95	40,00	29,20	34,71	40,00	33,08	31,89	27,66	23,67

hsa-miR-128a-002216	34,54	40,00	28,22	33,33	40,00	27,46	34,34	27,23	23,71
hsa-miR-23b-000400	28,72	35,47	29,55	33,53	40,00	28,21	34,46	27,25	23,79
hsa-miR-212-000515	32,35	31,96	26,68	32,74	25,53	26,41	34,47	27,87	23,81
hsa-miR-500-002428	32,23	40,00	28,01	33,24	37,47	27,40	32,99	27,78	23,84
hsa-miR-518f-002388	29,60	31,32	27,95	16,11	16,34	16,88	33,40	28,01	23,91
hsa-miR-625-002431	34,27	35,42	27,13	35,46	40,00	28,92	34,15	28,79	24,05
hsa-miR-452-002329	40,00	40,00	40,00	40,00	40,00	40,00	40,00	29,67	24,06
hsa-miR-100-000437	32,27	40,00	28,53	35,09	40,00	27,52	35,58	28,19	24,17
hsa-miR-148b-000471	33,19	28,84	29,31	32,71	40,00	28,79	32,48	28,50	24,18
hsa-miR-424-000604	40,00	40,00	31,80	34,00	40,00	30,81	34,28	28,33	24,41
hsa-miR-671-3p-002322	33,37	40,00	28,88	35,20	40,00	26,95	34,24	29,56	24,47
hsa-miR-339-3p-002184	32,06	40,00	28,75	34,54	40,00	29,49	40,00	29,07	24,48
hsa-miR-302a-000529	19,74	18,21	18,20	18,01	18,66	17,83	24,65	24,69	24,53
hsa-miR-328-000543	32,72	35,27	28,11	33,02	35,36	26,93	35,35	27,61	24,56
hsa-miR-501-001047	40,00	40,00	27,38	30,99	40,00	26,46	34,23	28,05	24,73
hsa-miR-192-000491	33,25	40,00	29,81	35,41	33,54	30,43	34,44	29,27	24,73
hsa-miR-324-5p-000539	32,75	34,62	29,86	31,83	40,00	29,07	32,75	27,93	24,80
hsa-miR-32-002109	21,31	40,00	35,29	40,00	40,00	31,62	35,36	30,02	24,83
hsa-miR-99a-000435	33,01	40,00	28,15	34,50	35,11	27,15	34,22	28,97	24,85
hsa-miR-361-000554	40,00	40,00	29,22	34,58	40,00	28,91	35,54	28,38	24,98
hsa-miR-628-5p-002433	18,72	21,13	33,47	35,22	26,63	29,73	35,06	30,93	25,03
hsa-miR-22-000398	29,58	40,00	31,26	40,00	40,00	30,91	31,02	30,49	25,12
hsa-miR-200c-002300	33,74	40,00	29,95	34,47	40,00	29,67	33,42	29,95	25,30
hsa-miR-642-001592	33,31	40,00	27,43	23,44	27,25	22,49	37,12	29,29	25,33
hsa-miR-107-000443	40,00	40,00	30,06	33,28	40,00	29,92	34,90	29,75	25,47
mmu-miR-491-001630	32,78	40,00	29,46	40,00	40,00	31,57	33,38	29,59	25,55
hsa-miR-146b-3p-002361	34,96	40,00	29,04	36,72	40,00	28,69	34,99	29,80	25,57
hsa-miR-125b-000449	34,24	40,00	29,21	33,24	38,56	28,87	35,03	29,55	25,58
hsa-miR-579-002398	35,22	40,00	30,54	40,00	40,00	34,91	35,55	29,77	25,59
hsa-miR-148a-000470	35,31	40,00	30,06	34,06	40,00	29,31	33,24	30,03	25,60
hsa-miR-149-002255	40,00	40,00	30,01	40,00	40,00	29,14	34,62	29,94	25,64
hsa-miR-362-3p-002117	34,98	40,00	33,24	34,57	40,00	30,30	40,00	29,39	25,92
hsa-miR-95-000433	40,00	40,00	30,42	34,14	40,00	29,01	35,53	30,20	25,96
hsa-let-7c-000379	34,75	35,50	29,76	34,41	40,00	29,38	33,28	29,40	25,98
hsa-miR-449-001030	35,40	40,00	31,57	34,97	40,00	32,99	35,45	30,81	25,99
hsa-miR-135a-000460	33,83	40,00	30,53	33,10	38,05	29,41	34,51	30,33	26,02
hsa-miR-423-5p-002340	34,12	40,00	28,24	33,19	40,00	28,73	33,33	29,04	26,04
hsa-miR-545-002267	40,00	40,00	32,00	40,00	40,00	29,78	34,18	30,15	26,04
mmu-miR-615-001960	40,00	40,00	28,14	40,00	40,00	27,15	40,00	29,05	26,15
hsa-miR-9-000583	34,88	40,00	29,96	35,23	40,00	29,31	34,50	30,78	26,26
hsa-miR-874-002268	40,00	9,14	31,79	40,00	40,00	30,10	35,26	30,28	26,50
hsa-miR-502-3p-002083	34,49	40,00	30,18	40,00	40,00	30,76	35,49	30,35	26,52
hsa-miR-181a-000480	34,26	40,00	30,00	33,70	40,00	29,95	34,50	31,14	26,71
hsa-miR-342-5p-002147	40,00	40,00	28,16	18,33	20,73	18,78	35,42	30,41	26,73
hsa-miR-130a-000454	34,71	40,00	32,11	33,88	40,00	32,85	34,70	31,27	26,93
hsa-miR-542-5p-002240	40,00	40,00	31,84	40,00	40,00	31,90	40,00	31,19	27,11
hsa-miR-126-002228	35,32	40,00	32,50	35,44	40,00	31,58	36,10	31,79	27,21
hsa-miR-636-002088	34,88	26,11	32,29	33,06	35,72	30,36	32,24	31,42	27,26
hsa-miR-629-002436	34,99	40,00	30,51	40,00	40,00	31,94	34,98	30,91	27,41
hsa-miR-194-000493	35,45	40,00	31,29	33,25	40,00	30,51	40,00	31,58	27,43

hsa-miR-501-3p-002435	40,00	40,00	30,83	40,00	40,00	31,33	32,92	30,10	27,45
hsa-miR-615-5p-002353	40,00	40,00	34,06	40,00	40,00	31,03	40,00	33,87	27,45
hsa-miR-450b-5p-002207	40,00	40,00	33,38	40,00	40,00	34,71	35,29	31,64	27,46
hsa-miR-523-002386	25,37	27,05	25,81	22,46	29,08	18,00	28,99	26,58	27,47
hsa-miR-505-002089	40,00	40,00	31,24	40,00	40,00	29,62	40,00	30,83	27,50
hsa-miR-449b-001608	33,29	40,00	31,87	34,64	38,50	31,70	33,26	32,60	27,66
hsa-miR-152-000475	21,41	40,00	32,29	35,56	27,07	32,58	40,00	31,37	27,70
mmu-miR-96-000186	40,00	40,00	32,38	35,28	40,00	29,83	40,00	34,52	27,77
hsa-miR-548d-5p-002237	40,00	40,00	32,15	40,00	40,00	32,55	35,55	30,99	27,78
hsa-miR-150-000473	40,00	40,00	31,66	40,00	40,00	31,28	35,31	33,20	27,94
hsa-miR-199a-3p-002304	40,00	40,00	33,17	40,00	40,00	30,95	34,76	31,63	27,95
hsa-miR-576-3p-002351	40,00	40,00	33,65	29,33	35,78	32,19	37,05	32,93	28,06
hsa-miR-133b-002247	40,00	40,00	32,40	34,85	40,00	30,44	40,00	32,54	28,07
hsa-miR-330-5p-002230	40,00	40,00	34,88	34,67	40,00	30,99	35,87	34,01	28,31
hsa-miR-589-002409	34,98	40,00	31,28	37,17	40,00	33,17	40,00	32,60	28,35
hsa-miR-891a-002191	40,00	40,00	34,69	40,00	40,00	33,98	34,73	32,87	28,38
hsa-miR-548b-5p-002408	34,20	40,00	32,96	40,00	40,00	31,08	34,65	32,19	28,38
hsa-miR-331-5p-002233	40,00	40,00	35,00	21,91	8,29	31,39	35,12	34,39	28,39
hsa-miR-299-5p-000600	26,21	40,00	31,91	20,22	22,99	20,48	27,31	27,63	28,43
hsa-miR-133a-002246	40,00	40,00	33,13	40,00	40,00	32,64	35,58	33,84	28,68
hsa-miR-223-002295	34,45	34,98	32,02	21,99	33,13	30,82	35,48	32,46	28,84
hsa-miR-542-3p-001284	35,50	40,00	35,54	40,00	40,00	34,02	40,00	32,37	28,87
hsa-miR-10a-000387	40,00	40,00	31,76	34,88	40,00	31,85	40,00	32,86	28,91
hsa-miR-450a-002303	40,00	40,00	33,97	34,50	40,00	32,39	35,43	32,42	28,96
hsa-miR-597-001551	34,42	34,78	33,21	35,49	35,95	32,82	34,26	32,28	28,97
hsa-miR-139-5p-002289	33,99	40,00	31,32	40,00	40,00	31,08	40,00	32,29	28,97
hsa-miR-296-000527	40,00	40,00	31,88	35,29	34,63	28,11	40,00	32,35	29,12
hsa-miR-616-002414	40,00	40,00	33,18	40,00	40,00	35,45	35,47	32,39	29,17
hsa-miR-502-001109	40,00	40,00	32,47	34,34	40,00	31,46	35,41	32,47	29,38
hsa-miR-219-1-3p-002095	40,00	40,00	34,03	40,00	40,00	34,99	40,00	33,19	29,39
hsa-miR-627-001560	33,00	18,07	40,00	40,00	40,00	35,55	35,46	34,06	29,48
hsa-miR-548c-5p-002429	38,47	40,00	34,26	40,00	40,00	32,41	40,00	33,87	29,60
hsa-miR-888-002212	17,34	18,27	18,02	23,43	25,17	35,27	20,30	33,63	29,63
hsa-miR-372-000560	31,60	32,13	32,11	40,00	40,00	40,00	33,90	30,15	29,68
hsa-miR-503-001048	34,10	40,00	32,79	35,11	27,24	33,22	40,00	31,92	29,95
hsa-miR-205-000509	40,00	40,00	25,73	40,00	40,00	40,00	40,00	33,63	29,98
hsa-miR-548d-001605	40,00	40,00	35,01	40,00	40,00	35,47	40,00	34,36	30,14
hsa-miR-190-000489	38,69	40,00	35,44	33,71	40,00	34,13	40,00	33,81	30,25
hsa-miR-219-000522	40,00	40,00	40,00	40,00	40,00	33,77	40,00	33,23	30,28
hsa-miR-494-002365	29,52	32,27	30,74	31,89	35,38	34,49	30,31	29,86	30,30
mmu-miR-153-001191	40,00	40,00	40,00	40,00	40,00	33,45	40,00	35,36	30,35
hsa-miR-141-000463	40,00	40,00	33,90	37,29	40,00	34,44	35,97	34,33	30,38
hsa-miR-138-002284	40,00	40,00	35,01	40,00	40,00	33,94	40,00	33,69	30,39
hsa-miR-214-002306	40,00	40,00	34,46	40,00	40,00	34,12	40,00	33,52	30,40
hsa-miR-574-3p-002349	36,74	40,00	34,42	35,32	40,00	33,40	35,43	35,03	30,40
mmu-miR-499-001352	40,00	40,00	32,80	40,00	40,00	35,28	40,00	34,63	30,45
hsa-miR-886-5p-002193	40,00	40,00	36,28	40,00	40,00	35,08	37,63	33,82	30,51

hsa-miR-576-5p-002350	40,00	40,00	34,57	40,00	40,00	35,34	40,00	34,97	30,60
hsa-miR-570-002347	40,00	40,00	40,00	40,00	40,00	35,35	40,00	35,08	30,61
hsa-miR-33b-002085	40,00	40,00	40,00	24,29	36,39	18,18	40,00	33,42	30,99
mmu-miR-124a-001182	31,59	34,91	33,14	31,32	40,00	34,26	33,61	31,72	31,16
hsa-miR-215-000518	40,00	40,00	30,97	34,27	7,72	30,83	36,67	33,05	31,17
hsa-miR-200b-002251	40,00	40,00	33,90	40,00	40,00	33,19	40,00	34,48	31,44
hsa-miR-181c-000482	40,00	40,00	33,32	40,00	40,00	33,42	40,00	35,92	31,67
hsa-miR-382-000572	40,00	40,00	40,00	35,00	40,00	40,00	40,00	40,00	31,75
hsa-miR-885-5p-002296	33,29	40,00	33,65	32,96	40,00	32,30	34,76	33,93	31,85
hsa-miR-490-001037	40,00	40,00	33,40	40,00	36,94	34,05	40,00	40,00	31,96
hsa-miR-105-002167	40,00	40,00	37,55	40,00	40,00	34,37	40,00	40,00	32,01
hsa-miR-483-5p-002338	35,42	40,00	40,00	35,36	40,00	33,53	36,15	32,58	32,16
hsa-miR-381-000571	28,86	40,00	40,00	35,16	35,26	35,13	31,35	31,89	32,24
hsa-miR-302b-000531	35,34	29,39	35,17	40,00	40,00	35,22	40,00	35,50	32,30
hsa-miR-145-002278	40,00	40,00	33,59	40,00	40,00	34,77	40,00	35,35	32,30
hsa-miR-203-000507	40,00	40,00	40,00	40,00	35,37	33,20	40,00	34,86	32,34
hsa-miR-184-000485	40,00	35,12	35,27	30,98	38,01	34,65	40,00	40,00	32,41
hsa-miR-375-000564	15,75	40,00	35,15	30,23	35,33	33,06	40,00	40,00	32,56
hsa-miR-548a-001538	40,00	40,00	35,23	40,00	40,00	40,00	40,00	40,00	32,68
hsa-miR-548b-001541	40,00	40,00	27,85	23,31	27,45	21,70	40,00	40,00	32,70
mmu-miR-187-001193	40,00	40,00	40,00	40,00	40,00	40,00	40,00	40,00	32,72
mmu-miR-137-001129	34,53	40,00	26,37	40,00	40,00	40,00	40,00	40,00	32,77
hsa-miR-199a-000498	40,00	40,00	40,00	40,00	40,00	40,00	40,00	36,02	32,81
mmu-miR-129-3p-001184	40,00	34,22	35,07	40,00	40,00	35,28	40,00	33,83	32,87
hsa-miR-200a-000502	40,00	40,00	34,07	40,00	40,00	30,64	40,00	33,89	32,99
hsa-miR-188-3p-002106	40,00	40,00	40,00	40,00	40,00	40,00	40,00	34,07	33,04
hsa-miR-518d-001159	32,42	40,00	40,00	34,38	40,00	40,00	33,29	40,00	33,28
hsa-miR-548a-5p-002412	40,00	13,84	36,51	40,00	40,00	40,00	40,00	40,00	33,37
hsa-miR-598-001988	40,00	40,00	40,00	40,00	40,00	40,00	40,00	40,00	33,49
hsa-miR-204-000508	40,00	40,00	34,75	20,24	21,05	35,05	40,00	35,32	33,51
hsa-miR-367-000555	40,00	40,00	37,32	40,00	40,00	35,49	40,00	40,00	33,56
hsa-miR-887-002374	40,00	40,00	36,49	40,00	23,25	31,48	40,00	40,00	33,72
mmu-miR-495-001663	40,00	40,00	40,00	40,00	40,00	40,00	40,00	40,00	33,90
hsa-miR-346-000553	40,00	31,93	31,96	33,74	35,35	31,88	34,15	40,00	33,95
hsa-miR-1-002222	40,00	40,00	30,05	33,12	40,00	26,17	40,00	40,00	33,95
hsa-miR-323-3p-002227	32,92	33,40	32,41	40,00	40,00	18,19	33,97	33,45	34,08
hsa-miR-296-3p-002101	40,00	40,00	40,00	40,00	40,00	35,75	40,00	40,00	34,13
hsa-miR-202-002363	33,97	35,52	34,45	40,00	40,00	40,00	35,27	35,13	34,14
hsa-miR-99b-000436	40,00	40,00	40,00	40,00	40,00	34,73	40,00	40,00	34,15
hsa-miR-34c-000428	40,00	40,00	40,00	40,00	40,00	40,00	40,00	40,00	34,16
hsa-miR-517a-002402	40,00	40,00	34,76	40,00	40,00	40,00	40,00	40,00	34,20
hsa-miR-512-3p-001823	40,00	40,00	40,00	40,00	36,72	40,00	40,00	40,00	34,22
hsa-miR-410-001274	40,00	40,00	28,90	40,00	40,00	40,00	40,00	40,00	34,33
hsa-miR-429-001024	40,00	40,00	35,47	40,00	40,00	40,00	40,00	35,19	34,37
hsa-miR-147b-002262	40,00	40,00	40,00	40,00	40,00	27,35	40,00	40,00	34,39
hsa-miR-582-3p-002399	35,64	40,00	33,37	40,00	14,27	40,00	40,00	40,00	34,40
hsa-miR-892a-002195	40,00	40,00	40,00	40,00	40,00	40,00	40,00	40,00	34,42
mmu-miR-134-001186	40,00	40,00	40,00	40,00	40,00	40,00	40,00	40,00	34,50
hsa-miR-326-000542	40,00	40,00	40,00	40,00	40,00	40,00	40,00	40,00	34,51

hsa-miR-143-002249	36,80	40,00	40,00	40,00	40,00	40,00	40,00	40,00	34,56
hsa-miR-890-002209	40,00	40,00	40,00	40,00	40,00	40,00	40,00	40,00	34,56
hsa-miR-582-5p-001983	40,00	40,00	40,00	40,00	40,00	33,81	40,00	40,00	34,57
hsa-miR-211-000514	40,00	40,00	40,00	40,00	40,00	40,00	40,00	36,06	34,63
hsa-miR-376c-002122	40,00	40,00	40,00	40,00	40,00	40,00	40,00	34,98	34,65
hsa-miR-655-001612	40,00	40,00	40,00	40,00	40,00	40,00	40,00	36,62	34,70
hsa-miR-455-001280	40,00	40,00	40,00	40,00	40,00	40,00	40,00	34,55	34,73
hsa-miR-193a-3p-002250	40,00	40,00	40,00	40,00	40,00	40,00	40,00	40,00	34,74
hsa-miR-520g-001121	40,00	40,00	40,00	40,00	40,00	40,00	40,00	40,00	34,81
hsa-miR-518b-001156	40,00	40,00	40,00	24,20	30,36	23,35	40,00	40,00	34,83
hsa-miR-193a-5p-002281	40,00	40,00	40,00	40,00	40,00	40,00	40,00	40,00	34,83
hsa-miR-511-001111	40,00	40,00	40,00	22,90	40,00	18,59	40,00	40,00	34,90
hsa-miR-218-000521	20,83	40,00	36,46	19,29	20,57	19,67	25,04	35,05	34,99
hsa-miR-302c-000533	40,00	32,45	31,54	40,00	40,00	40,00	40,00	40,00	35,03
hsa-miR-216b-002326	40,00	40,00	40,00	40,00	40,00	40,00	40,00	40,00	35,05
hsa-miR-491-3p-002360	40,00	40,00	40,00	40,00	40,00	40,00	40,00	35,43	35,07
hsa-miR-518d-5p-002389	40,00	40,00	40,00	40,00	40,00	40,00	40,00	40,00	35,08
hsa-miR-519e-002370	40,00	40,00	40,00	40,00	40,00	40,00	34,85	40,00	35,08
hsa-miR-10b-002218	40,00	40,00	40,00	40,00	40,00	40,00	40,00	40,00	35,13
hsa-miR-488-002357	40,00	40,00	40,00	40,00	40,00	40,00	40,00	40,00	35,16
hsa-miR-891b-002210	40,00	40,00	40,00	40,00	40,00	40,00	40,00	40,00	35,17
hsa-miR-519a-002415	33,99	35,60	34,35	20,20	40,00	18,83	40,00	40,00	35,20
hsa-miR-433-001028	34,32	40,00	40,00	35,28	40,00	40,00	34,57	40,00	35,25
hsa-miR-217-002337	40,00	40,00	25,72	40,00	40,00	40,00	40,00	40,00	35,31
hsa-miR-199b-000500	40,00	40,00	40,00	29,70	40,00	40,00	40,00	40,00	35,32
hsa-miR-383-000573	40,00	40,00	40,00	40,00	40,00	40,00	40,00	40,00	35,33
mmu-miR-451-001141	40,00	40,00	33,55	40,00	40,00	40,00	40,00	40,00	35,33
hsa-miR-618-001593	33,65	32,87	33,90	29,54	35,27	22,11	33,74	33,80	35,37
hsa-miR-125a-5p-002198	40,00	40,00	40,00	40,00	40,00	35,25	26,73	40,00	35,38
hsa-miR-517c-001153	28,56	40,00	40,00	40,00	19,16	40,00	40,00	40,00	35,45
hsa-miR-376a-000565	40,00	40,00	40,00	32,27	34,98	35,08	40,00	40,00	35,45
hsa-miR-411-001610	40,00	40,00	34,97	33,02	40,00	35,51	40,00	40,00	35,48
hsa-miR-224-002099	40,00	40,00	40,00	40,00	9,82	40,00	40,00	40,00	35,51
hsa-miR-624-002430	40,00	40,00	40,00	40,00	40,00	40,00	40,00	40,00	35,57
hsa-miR-384-000574	34,41	40,00	40,00	27,38	29,22	23,37	40,00	35,75	36,00
hsa-miR-335-000546	40,00	40,00	40,00	40,00	40,00	40,00	40,00	40,00	36,30
hsa-miR-522-002413	32,67	37,10	33,15	40,00	40,00	40,00	34,29	40,00	36,97
hsa-miR-548c-001590	40,00	40,00	40,00	40,00	40,00	40,00	40,00	40,00	39,10
hsa-miR-489-002358	18,50	29,98	19,70	19,97	22,79	20,76	34,32	40,00	40,00
hsa-miR-136-000592	40,00	40,00	31,30	32,57	34,49	22,59	40,00	40,00	40,00
hsa-miR-561-001528	40,00	40,00	40,00	17,53	40,00	17,78	40,00	40,00	40,00
hsa-miR-872-002264	40,00	40,00	40,00	19,57	24,21	18,23	40,00	40,00	40,00
hsa-miR-876-5p-002205	40,00	40,00	40,00	18,92	22,31	18,59	40,00	40,00	40,00
hsa-miR-520e-001119	40,00	28,40	27,51	31,63	33,09	31,66	40,00	40,00	40,00
hsa-miR-518e-002395	40,00	18,85	19,25	37,12	40,00	40,00	40,00	40,00	40,00
hsa-miR-198-002273	40,00	40,00	40,00	26,30	40,00	20,50	37,49	40,00	40,00
hsa-miR-520f-001120	40,00	40,00	40,00	24,82	35,41	20,74	40,00	40,00	40,00
hsa-miR-329-001101	40,00	40,00	40,00	26,77	40,00	21,06	40,00	40,00	40,00

hsa-miR-219-2-3p-002390	40,00	31,81	21,55	40,00	40,00	40,00	40,00	40,00	40,00
hsa-miR-520b-001116	40,00	40,00	40,00	21,54	40,00	21,56	38,44	40,00	40,00
hsa-miR-520a-001167	40,00	40,00	40,00	23,46	27,37	22,09	40,00	40,00	40,00
hsa-miR-376b-001102	40,00	40,00	40,00	24,07	28,40	23,31	40,00	40,00	40,00
hsa-miR-337-5p-002156	35,25	40,00	40,00	25,89	32,64	23,36	40,00	40,00	40,00
hsa-miR-513-5p-002090	40,00	40,00	38,97	32,00	40,00	24,40	40,00	40,00	40,00
hsa-miR-512-5p-001145	40,00	40,00	40,00	27,17	31,95	24,06	40,00	40,00	40,00
hsa-miR-125a-3p-002199	40,00	40,00	40,00	40,00	40,00	24,98	40,00	40,00	40,00
hsa-miR-492-001039	40,00	40,00	40,00	27,63	40,00	25,12	40,00	40,00	40,00
hsa-miR-455-3p-002244	40,00	40,00	40,00	40,00	40,00	25,91	40,00	40,00	40,00
hsa-miR-516b-001150	40,00	40,00	40,00	28,67	7,76	26,21	40,00	40,00	40,00
hsa-miR-519c-001163	40,00	40,00	26,32	40,00	40,00	40,00	40,00	40,00	40,00
hsa-miR-369-3p-000557	40,00	40,00	28,74	40,00	40,00	40,00	40,00	40,00	40,00
hsa-miR-672-002327	40,00	40,00	40,00	40,00	40,00	29,14	40,00	40,00	40,00
hsa-miR-450b-3p-002208	40,00	40,00	40,00	36,00	40,00	29,35	40,00	40,00	40,00
hsa-miR-147-000469	40,00	40,00	40,00	40,00	40,00	29,98	40,00	40,00	40,00
hsa-miR-363-001271	40,00	40,00	35,27	35,19	40,00	35,08	40,00	40,00	40,00
hsa-miR-31-002279	40,00	40,00	30,60	40,00	40,00	40,00	40,00	40,00	40,00
hsa-miR-509-5p-002235	35,03	35,55	35,44	35,34	37,23	35,54	40,00	35,12	40,00
hsa-miR-487a-001279	32,90	40,00	31,27	32,80	40,00	40,00	40,00	40,00	40,00
hsa-miR-370-002275	40,00	40,00	40,00	29,67	40,00	32,40	40,00	40,00	40,00
hsa-miR-551b-001535	40,00	40,00	40,00	34,27	35,22	32,45	40,00	40,00	40,00
hsa-miR-373-000561	34,72	40,00	33,02	40,00	40,00	40,00	40,00	40,00	40,00
hsa-miR-122-002245	40,00	40,00	40,00	40,00	40,00	33,94	40,00	40,00	40,00
hsa-miR-139-3p-002313	40,00	40,00	34,03	34,11	40,00	40,00	40,00	40,00	40,00
hsa-miR-485-3p-001277	33,72	36,45	34,65	34,98	34,42	40,00	40,00	40,00	40,00
hsa-miR-539-001286	40,00	40,00	40,00	40,00	39,28	34,87	40,00	40,00	40,00
hsa-miR-371-3p-002124	40,00	40,00	35,43	40,00	40,00	40,00	40,00	40,00	40,00
hsa-miR-127-000452	40,00	40,00	40,00	40,00	40,00	40,00	40,00	40,00	40,00
hsa-miR-127-5p-002229	40,00	40,00	40,00	40,00	40,00	40,00	40,00	40,00	40,00
hsa-miR-129-000590	40,00	40,00	40,00	23,29	40,00	40,00	40,00	40,00	40,00
hsa-miR-154-000477	40,00	40,00	40,00	40,00	40,00	40,00	40,00	40,00	40,00
hsa-miR-208-000511	40,00	40,00	40,00	40,00	40,00	40,00	34,25	40,00	40,00
hsa-miR-208b-002290	40,00	40,00	40,00	40,00	40,00	40,00	40,00	40,00	40,00
hsa-miR-216a-002220	40,00	40,00	40,00	40,00	40,00	40,00	40,00	40,00	40,00
hsa-miR-220-000523	40,00	40,00	40,00	40,00	40,00	40,00	40,00	40,00	40,00
hsa-miR-220b-002206	40,00	40,00	40,00	40,00	40,00	40,00	40,00	40,00	40,00
hsa-miR-220c-002211	40,00	40,00	40,00	40,00	40,00	40,00	40,00	40,00	40,00
hsa-miR-298-002190	40,00	40,00	40,00	40,00	40,00	40,00	40,00	40,00	40,00
hsa-miR-299-3p-001015	35,19	40,00	40,00	40,00	40,00	40,00	40,00	38,13	40,00
hsa-miR-325-000540	40,00	40,00	40,00	40,00	40,00	40,00	40,00	40,00	40,00
hsa-miR-338-3p-002252	40,00	40,00	40,00	40,00	40,00	40,00	40,00	40,00	40,00
hsa-miR-340-002258	40,00	40,00	40,00	40,00	40,00	40,00	40,00	40,00	40,00
hsa-miR-369-5p-001021	40,00	40,00	40,00	40,00	40,00	40,00	40,00	40,00	40,00
hsa-miR-377-000566	40,00	40,00	40,00	40,00	40,00	40,00	40,00	40,00	40,00
hsa-miR-380-3p-000569	40,00	40,00	40,00	40,00	40,00	40,00	40,00	40,00	40,00
hsa-miR-409-5p-002331	40,00	40,00	40,00	40,00	40,00	40,00	40,00	40,00	40,00
hsa-miR-412-001023	40,00	40,00	40,00	40,00	40,00	40,00	40,00	40,00	40,00
hsa-miR-431-001979	40,00	40,00	40,00	40,00	40,00	40,00	40,00	40,00	40,00

hsa-miR-448-001029	40,00	40,00	40,00	40,00	40,00	40,00	40,00	40,00	40,00
hsa-miR-453-002318	40,00	40,00	40,00	40,00	40,00	40,00	40,00	40,00	40,00
hsa-miR-485-5p-001036	40,00	40,00	40,00	40,00	40,00	40,00	40,00	40,00	40,00
hsa-miR-487b-001285	40,00	40,00	40,00	40,00	40,00	40,00	40,00	40,00	40,00
hsa-miR-493-002364	40,00	40,00	40,00	40,00	40,00	40,00	40,00	40,00	40,00
hsa-miR-499-3p-002427	40,00	40,00	40,00	40,00	40,00	40,00	40,00	40,00	40,00
hsa-miR-504-002084	40,00	40,00	40,00	40,00	40,00	40,00	40,00	40,00	40,00
hsa-miR-506-001050	40,00	40,00	40,00	40,00	40,00	40,00	40,00	40,00	40,00
hsa-miR-507-001051	35,22	40,00	40,00	40,00	40,00	40,00	40,00	40,00	40,00
hsa-miR-508-5p-002092	40,00	40,00	40,00	40,00	40,00	40,00	40,00	40,00	40,00
hsa-miR-509-3-5p-002155	40,00	40,00	40,00	40,00	40,00	40,00	40,00	40,00	40,00
hsa-miR-510-002241	40,00	40,00	40,00	40,00	40,00	40,00	40,00	40,00	40,00
hsa-miR-515-3p-002369	40,00	40,00	40,00	40,00	40,00	40,00	37,96	40,00	40,00
hsa-miR-515-5p-001112	40,00	40,00	40,00	40,00	40,00	40,00	40,00	40,00	40,00
hsa-miR-516a-5p-002416	40,00	40,00	40,00	40,00	32,39	40,00	40,00	40,00	40,00
hsa-miR-517b-001152	34,80	40,00	40,00	36,04	40,00	40,00	34,39	40,00	40,00
hsa-miR-518a-3p-002397	40,00	40,00	40,00	40,00	40,00	40,00	40,00	40,00	40,00
hsa-miR-518a-5p-002396	40,00	40,00	40,00	40,00	40,00	40,00	40,00	40,00	40,00
hsa-miR-518c-002401	40,00	40,00	40,00	40,00	40,00	40,00	40,00	40,00	40,00
hsa-miR-520a#-001168	40,00	40,00	40,00	36,61	40,00	40,00	40,00	40,00	40,00
hsa-miR-520d-5p-002393	40,00	40,00	40,00	40,00	40,00	40,00	40,00	39,56	40,00
hsa-miR-521-001122	40,00	40,00	40,00	40,00	40,00	40,00	40,00	40,00	40,00
hsa-miR-524-5p-001982	40,00	40,00	40,00	40,00	40,00	40,00	40,00	40,00	40,00
hsa-miR-525-001174	40,00	40,00	40,00	40,00	40,00	40,00	40,00	40,00	40,00
hsa-miR-526b-002382	40,00	40,00	40,00	40,00	40,00	40,00	40,00	40,00	40,00
hsa-miR-541-002201	40,00	40,00	40,00	40,00	40,00	40,00	40,00	40,00	40,00
hsa-miR-544-002265	40,00	40,00	40,00	40,00	40,00	40,00	40,00	40,00	40,00
hsa-miR-556-3p-002345	40,00	40,00	40,00	40,00	40,00	40,00	40,00	40,00	40,00
hsa-miR-556-5p-002344	40,00	40,00	40,00	40,00	40,00	40,00	40,00	40,00	40,00
hsa-miR-651-001604	40,00	40,00	40,00	40,00	40,00	40,00	40,00	40,00	40,00
hsa-miR-653-002292	40,00	40,00	40,00	40,00	40,00	40,00	40,00	40,00	40,00
hsa-miR-654-001611	40,00	40,00	40,00	40,00	40,00	40,00	40,00	40,00	40,00
hsa-miR-654-3p-002239	40,00	40,00	40,00	40,00	40,00	40,00	40,00	40,00	40,00
hsa-miR-674-002021	40,00	40,00	40,00	40,00	40,00	40,00	40,00	40,00	40,00
hsa-miR-758-001990	40,00	40,00	40,00	40,00	40,00	40,00	40,00	40,00	40,00
hsa-miR-871-002354	40,00	40,00	40,00	40,00	40,00	40,00	40,00	40,00	40,00
hsa-miR-873-002356	40,00	40,00	40,00	40,00	40,00	40,00	40,00	40,00	40,00
hsa-miR-875-3p-002204	40,00	40,00	40,00	40,00	40,00	40,00	40,00	40,00	40,00
hsa-miR-876-3p-002225	30,88	22,27	40,00	40,00	40,00	40,00	40,00	40,00	40,00
hsa-miR-885-3p-002372	40,00	40,00	40,00	40,00	40,00	40,00	40,00	40,00	40,00
hsa-miR-886-3p-002194	40,00	40,00	40,00	38,55	40,00	40,00	38,76	33,97	40,00
hsa-miR-889-002202	40,00	40,00	40,00	40,00	40,00	40,00	40,00	40,00	40,00
mmu-miR-379-001138	33,21	40,00	40,00	34,81	40,00	40,00	40,00	35,02	40,00
mmu-miR-496-001953	35,40	40,00	40,00	40,00	40,00	40,00	40,00	40,00	40,00

fold enrichment

	Ago2 IP/TL	IgG IP/TL	Ago2IP/IgG IP
hsa-miR-486-3p-002093	9574,62	5,85	1637,56
mmu-miR-615-001960	7396,70	12,57	588,54
hsa-miR-508-001052	3404,75	8,43	403,85
hsa-miR-30b-000602	2374,38	7,02	338,42
hsa-miR-27a-000408	1711,05	10,37	164,93
hsa-miR-505-002089	1496,46	8,32	179,89
hsa-miR-149-002255	982,51	2,94	333,84
hsa-miR-29c-000587	914,81	2,59	353,28
hsa-miR-542-5p-002240	843,36	7,65	110,23
hsa-miR-140-3p-002234	634,00	1,75	362,04
hsa-miR-339-5p-002257	577,76	2,26	256,18
hsa-miR-615-5p-002353	568,89	4,12	138,05
hsa-miR-342-3p-002260	527,49	0,59	889,59
hsa-miR-361-000554	513,18	1,50	343,14
hsa-miR-874-002268	496,96	3947,34	0,13
hsa-miR-365-001020	484,38	1,14	424,71
hsa-miR-501-001047	471,90	0,52	907,87
hsa-miR-29b-000413	449,24	0,48	935,98
hsa-miR-545-002267	441,62	2,54	173,85
hsa-miR-296-000527	423,53	6,82	62,14
hsa-miR-28-3p-002446	396,18	2,15	184,14
hsa-miR-34a-000426	357,80	4,82	74,27
mmu-miR-96-000186	345,61	1,19	290,09
hsa-miR-222-002276	319,35	0,38	834,44
hsa-miR-532-001518	317,07	0,87	366,16
hsa-miR-150-000473	282,41	1,63	173,49
hsa-miR-345-002186	273,61	0,31	868,87
hsa-miR-205-000509	273,42	4,35	62,80
hsa-miR-95-000433	272,54	0,89	307,83
hsa-miR-362-001273	255,23	2,12	120,15
hsa-miR-133b-002247	252,71	1,70	148,23
hsa-miR-339-3p-002184	249,29	0,57	440,50
hsa-miR-532-3p-002355	244,44	0,95	256,65
hsa-miR-625-002431	243,43	0,93	262,77
hsa-miR-221-000524	236,71	0,13	1808,61
hsa-miR-197-000497	235,02	0,66	357,88
hsa-miR-320-002277	229,34	0,93	245,34
hsa-miR-186-002285	226,60	0,67	339,67
hsa-miR-146b-3p-002361	220,71	0,48	455,09
hsa-miR-484-001821	219,79	6,03	36,45
hsa-miR-501-3p-002435	218,63	1,92	114,09
hsa-miR-548d-5p-002237	206,45	2,87	71,97
hsa-miR-1-002222	200,30	0,20	982,51
hsa-miR-128a-002216	194,82	0,31	621,24
hsa-miR-107-000443	190,77	0,69	274,56
hsa-miR-100-000437	190,24	0,30	639,74
hsa-miR-199a-3p-002304	188,84	2,06	91,71

hsa-miR-212-000515	188,18	26,61	7,07
hsa-miR-139-5p-002289	186,24	1,48	125,77
hsa-miR-23a-000399	185,46	0,24	786,88
hsa-miR-502-3p-002083	181,82	0,92	197,86
hsa-miR-671-3p-002322	181,73	0,21	864,67
hsa-miR-331-000545	176,11	0,54	325,53
hsa-miR-10a-000387	175,30	1,60	109,90
hsa-miR-425-5p-001516	174,69	0,63	275,26
hsa-miR-200a-000502	172,69	4,10	42,09
hsa-let-7b-002619	172,53	0,13	1279,77
hsa-miR-323-3p-002227	169,56	1,01	167,89
hsa-miR-548c-5p-002429	168,86	2,89	58,36
hsa-miR-183-002269	157,66	0,11	1393,99
hsa-miR-708-002341	156,61	0,30	523,36
hsa-miR-652-002352	156,32	0,09	1744,58
hsa-miR-146a-000468	150,19	0,12	1301,84
hsa-miR-219-1-3p-002095	146,90	4,83	30,44
hsa-miR-191-002299	146,90	0,39	379,42
hsa-miR-99a-000435	146,63	0,58	251,89
hsa-miR-181c-000482	146,52	2,56	57,15
hsa-miR-328-000543	143,48	1,93	74,23
hsa-miR-330-000544	143,28	0,53	270,97
hsa-miR-200b-002251	143,05	3,58	39,97
mmu-miR-499-001352	142,85	3,45	41,36
hsa-miR-18b-002217	142,78	0,04	4047,08
hsa-miR-26a-000405	138,52	0,36	389,37
hsa-miR-424-000604	135,67	0,99	137,25
hsa-miR-193b-002367	132,09	0,53	247,17
hsa-miR-133a-002246	132,06	1,49	88,34
hsa-miR-422a-002297	131,75	1,97	67,01
mmu-miR-374-5p-001319	130,63	10,22	12,79
hsa-miR-548b-001541	129,70	0,38	337,25
hsa-miR-214-002306	128,77	4,47	28,79
hsa-miR-182-002334	128,12	0,20	625,12
hsa-miR-324-3p-002161	125,31	0,79	157,84
hsa-miR-486-001278	123,53	0,36	346,73
hsa-miR-16-000391	121,26	0,30	405,81
hsa-miR-138-002284	118,44	4,30	27,53
hsa-miR-490-001037	116,32	2,03	57,35
hsa-miR-27b-000409	115,76	0,06	1829,20
hsa-miR-130b-000456	115,39	0,03	3608,88
hsa-miR-374-000563	115,20	0,45	254,47
hsa-miR-132-000457	111,64	0,48	231,84
hsa-miR-146b-001097	108,84	0,28	390,09
hsa-miR-101-002253	107,19	0,27	396,63
hsa-miR-24-000402	104,64	0,47	222,96
hsa-miR-629-002436	104,40	0,81	129,67
hsa-miR-21-000397	104,11	0,21	507,06
hsa-miR-342-5p-002147	103,85	1,83	56,65
hsa-miR-362-3p-002117	103,75	1,04	99,99
hsa-miR-135b-002261	102,85	0,39	264,78

hsa-miR-196b-002215	100,75	0,15	659,24
hsa-miR-590-5p-001984	100,68	0,38	268,42
hsa-miR-103-000439	97,84	0,11	855,92
hsa-miR-20b-001014	96,56	0,53	180,73
hsa-miR-18a-002422	95,69	0,37	256,30
hsa-miR-450b-5p-002207	95,67	2,33	41,09
hsa-miR-579-002398	95,08	1,26	75,60
hsa-miR-25-000403	93,55	0,59	159,38
mmu-miR-491-001630	92,30	0,45	204,18
hsa-miR-576-5p-002350	90,30	3,20	28,22
hsa-miR-194-000493	90,03	0,51	175,10
hsa-miR-454-002323	89,49	0,32	275,84
hsa-miR-548d-001605	87,97	3,68	23,89
hsa-miR-589-002409	87,53	0,90	97,05
hsa-miR-195-000494	87,22	0,16	556,02
hsa-miR-145-002278	87,22	2,93	29,81
hsa-miR-15b-000390	86,66	0,10	879,78
hsa-miR-106b-000442	85,69	0,15	565,87
hsa-miR-500-002428	84,59	0,21	406,28
hsa-let-7d-002283	82,23	0,18	462,51
hsa-miR-9-000583	82,12	0,24	341,72
hsa-miR-301b-002392	80,49	0,14	565,61
hsa-miR-217-002337	80,08	1,00	80,08
**hsa-miR-155-002623**	**79,62**	**0,43**	**185,85**
hsa-miR-125b-000449	77,73	0,27	283,13
hsa-miR-660-001515	77,73	0,24	321,57
hsa-miR-20a-000580	76,59	0,23	336,86
hsa-let-7a-000377	76,13	0,10	796,02
hsa-miR-29a-002112	76,09	0,26	297,62
hsa-miR-136-000592	74,92	0,64	116,73
hsa-miR-642-001592	73,96	0,54	136,97
hsa-let-7g-002282	71,52	0,16	450,59
hsa-miR-219-2-3p-002390	71,08	6,63	10,72
hsa-miR-106a-002169	69,92	0,29	237,70
hsa-miR-887-002374	68,75	47,99	1,43
hsa-miR-147b-002262	67,99	1,00	67,99
hsa-let-7e-002406	67,68	0,27	247,05
hsa-miR-301-000528	67,66	0,12	576,83
hsa-miR-192-000491	66,21	1,07	61,82
hsa-miR-17-002308	65,84	0,31	210,84
hsa-miR-98-000577	64,91	0,27	237,10
hsa-miR-215-000518	63,73	1064,28	0,06
hsa-miR-518e-002395	62,11	67,99	0,91
hsa-miR-891a-002191	59,47	1,54	38,69
hsa-miR-616-002414	59,18	2,04	29,03
hsa-miR-423-5p-002340	58,89	0,14	408,82
hsa-miR-148a-000470	58,78	0,18	326,36
hsa-let-7f-000382	55,01	0,23	236,39
hsa-let-7c-000379	54,59	0,57	96,40
hsa-miR-744-002324	54,12	0,13	432,23
hsa-miR-142-5p-002248	54,07	0,65	82,98

hsa-miR-19b-000396	53,03	0,19	274,06
hsa-miR-542-3p-001284	51,71	2,06	25,08
mmu-miR-129-3p-001184	48,36	15,81	3,06
hsa-miR-410-001274	48,17	1,00	48,17
hsa-miR-200c-002300	47,54	0,15	325,23
hsa-miR-210-000512	45,69	0,11	414,05
hsa-miR-548b-5p-002408	44,64	0,46	96,51
hsa-miR-502-001109	44,57	0,53	83,50
hsa-miR-330-5p-002230	43,77	0,45	97,70
mmu-miR-153-001191	42,23	2,92	14,45
hsa-miR-148b-000471	41,31	1,27	32,45
hsa-miR-525-3p-002385	41,03	1,04	39,41
hsa-miR-105-002167	41,01	1,00	41,01
mmu-miR-140-001187	40,83	0,06	635,17
hsa-miR-452-002329	39,80	10,88	3,66
hsa-miR-219-000522	39,77	4,78	8,31
hsa-miR-142-3p-000464	38,99	0,10	379,60
hsa-miR-181a-000480	38,55	0,13	286,16
hsa-miR-886-5p-002193	38,07	2,41	15,81
hsa-miR-15a-000389	36,80	0,14	270,10
hsa-miR-126-002228	36,47	0,32	114,06
hsa-miR-30c-000419	36,46	0,11	317,95
hsa-miR-19a-000395	35,93	0,12	303,87
hsa-miR-135a-000460	35,79	0,20	177,91
mmu-miR-137-001129	35,01	0,28	123,93
hsa-miR-449-001030	34,00	0,32	107,81
hsa-miR-23b-000400	33,25	0,25	133,31
hsa-miR-33b-002085	32,86	0,28	117,54
hsa-miR-125a-3p-002199	32,18	1,00	32,18
hsa-miR-92a-000431	31,71	0,13	235,24
hsa-miR-519d-002403	31,46	0,37	84,60
hsa-miR-450a-002303	29,24	0,56	52,01
hsa-miR-141-000463	28,80	0,78	36,86
hsa-miR-203-000507	28,25	9,56	2,96
hsa-miR-455-3p-002244	25,94	1,00	25,94
hsa-miR-570-002347	25,62	3,12	8,22
hsa-miR-26b-000407	24,46	0,06	388,92
hsa-miR-519c-001163	23,62	1,00	23,62
hsa-miR-367-000555	23,29	1,00	23,29
hsa-miR-185-002271	23,11	0,15	150,64
hsa-miR-324-5p-000539	23,06	0,30	77,23
hsa-miR-302c-000533	22,28	5,72	3,90
hsa-miR-22-000398	21,67	0,10	212,90
hsa-miR-503-001048	21,32	10,18	2,09
hsa-miR-28-000411	20,75	0,04	482,04
hsa-miR-302b-000531	18,61	11,20	1,66
hsa-miR-190-000489	18,28	0,72	25,31
hsa-miR-576-3p-002351	17,87	0,58	30,68
hsa-miR-520e-001119	17,81	10,42	1,71
hsa-miR-548a-001538	16,33	1,00	16,33
hsa-miR-582-5p-001983	14,64	1,00	14,64

hsa-miR-130a-000454	13,93	0,16	88,01
hsa-miR-369-3p-000557	13,49	1,00	13,49
hsa-miR-99b-000436	13,06	1,00	13,06
mmu-miR-451-001141	13,03	1,00	13,03
hsa-miR-517a-002402	12,82	1,00	12,82
hsa-miR-672-002327	12,29	1,00	12,29
hsa-miR-518f-002388	11,00	2,22	4,96
hsa-miR-636-002088	10,73	4,96	2,16
hsa-miR-429-001024	10,46	3,04	3,44
hsa-miR-548a-5p-002412	10,37	421,29	0,02
hsa-miR-296-3p-002101	10,36	1,00	10,36
hsa-miR-346-000553	10,31	1,15	8,97
RNU48-001006	10,21	0,74	13,73
hsa-miR-147-000469	10,12	1,00	10,12
hsa-miR-449b-001608	9,99	0,10	98,86
hsa-miR-511-001111	8,78	0,02	456,67
hsa-miR-31-002279	8,78	1,00	8,78
hsa-miR-574-3p-002349	8,51	0,18	48,63
hsa-miR-597-001551	8,32	1,31	6,37
U6 snRNA-001973	7,52	2,95	2,55
hsa-miR-184-000485	7,38	0,61	12,11
hsa-miR-513-5p-002090	7,34	0,16	46,63
RNU44-001094	6,18	0,54	11,35
hsa-miR-582-3p-002399	6,16	139,46	0,04
mmu-miR-187-001193	5,37	1,00	5,37
hsa-miR-199a-000498	5,26	2,51	2,10
hsa-miR-411-001610	5,11	0,20	25,63
hsa-miR-188-3p-002106	5,00	3,94	1,27
hsa-miR-450b-3p-002208	4,65	0,40	11,71
hsa-miR-598-001988	4,50	1,00	4,50
mmu-miR-495-001663	4,09	1,00	4,09
hsa-miR-122-002245	4,05	1,00	4,05
hsa-miR-518b-001156	4,02	0,24	16,68
hsa-miR-34c-000428	3,85	1,00	3,85
hsa-miR-519a-002415	3,82	0,01	537,08
hsa-miR-512-3p-001823	3,81	2,13	1,78
hsa-miR-329-001101	3,74	0,05	79,51
hsa-miR-892a-002195	3,63	1,00	3,63
hsa-miR-618-001593	3,61	0,31	11,47
hsa-miR-523-002386	3,59	0,26	14,04
mmu-miR-134-001186	3,56	1,00	3,56
hsa-miR-326-000542	3,56	1,00	3,56
hsa-miR-890-002209	3,52	1,00	3,52
hsa-miR-211-000514	3,46	2,48	1,39
hsa-miR-376c-002122	3,44	3,19	1,08
hsa-miR-655-001612	3,40	2,18	1,56
hsa-miR-455-001280	3,38	3,53	0,96
hsa-miR-193a-3p-002250	3,37	1,00	3,37
hsa-miR-520g-001121	3,31	1,00	3,31
hsa-miR-193a-5p-002281	3,30	1,00	3,30
hsa-miR-539-001286	3,27	1,18	2,77

hsa-miR-216b-002326	3,14	1,00	3,14
hsa-miR-491-3p-002360	3,13	2,88	1,09
hsa-miR-32-002109	3,12	0,05	68,23
hsa-miR-518d-5p-002389	3,12	1,00	3,12
hsa-miR-10b-002218	3,08	1,00	3,08
hsa-miR-488-002357	3,06	1,00	3,06
hsa-miR-363-001271	3,06	0,33	9,30
hsa-miR-891b-002210	3,05	1,00	3,05
hsa-miR-383-000573	2,94	1,00	2,94
hsa-miR-371-3p-002124	2,88	1,00	2,88
hsa-miR-224-002099	2,82	1068,47	0,00
hsa-miR-624-002430	2,78	1,00	2,78
hsa-miR-152-000475	2,76	0,71	3,88
hsa-miR-520f-001120	2,57	0,09	29,69
hsa-miR-372-000560	2,36	2,11	1,12
hsa-miR-335-000546	2,35	1,00	2,35
hsa-miR-627-001560	2,21	43,61	0,05
hsa-miR-198-002273	2,14	0,02	90,45
hsa-miR-382-000572	2,12	0,32	6,73
hsa-miR-885-5p-002296	2,10	0,05	41,60
hsa-miR-512-5p-001145	2,06	0,33	6,20
hsa-miR-492-001039	1,79	0,06	31,13
hsa-miR-516b-001150	1,77	125,51	0,01
hsa-miR-384-000574	1,75	0,48	3,65
hsa-miR-331-5p-002233	1,68	27,52	0,06
hsa-miR-143-002249	1,68	0,48	3,52
mmu-miR-93-001090	1,67	0,01	309,19
hsa-miR-302a-000529	1,53	1,21	1,26
hsa-miR-551b-001535	1,52	0,80	1,90
hsa-miR-376a-000565	1,49	0,53	2,79
hsa-miR-373-000561	1,48	0,30	5,02
hsa-miR-520a-001167	1,37	0,41	3,38
hsa-miR-872-002264	1,36	0,34	3,98
hsa-miR-483-5p-002338	1,33	0,27	4,91
hsa-miR-548c-001590	1,23	1,00	1,23
hsa-miR-376b-001102	1,19	0,37	3,24
hsa-miR-628-5p-002433	1,19	10,81	0,11
hsa-miR-202-002363	1,16	0,72	1,61
hsa-miR-876-5p-002205	1,08	0,46	2,36
hsa-miR-223-002295	1,06	0,14	7,80
hsa-miR-139-3p-002313	1,02	0,26	3,97
hsa-miR-127-000452	1,00	1,00	1,00
hsa-miR-127-5p-002229	1,00	1,00	1,00
hsa-miR-154-000477	1,00	1,00	1,00
hsa-miR-208b-002290	1,00	1,00	1,00
hsa-miR-216a-002220	1,00	1,00	1,00
hsa-miR-220-000523	1,00	1,00	1,00
hsa-miR-220b-002206	1,00	1,00	1,00
hsa-miR-220c-002211	1,00	1,00	1,00
hsa-miR-298-002190	1,00	1,00	1,00
hsa-miR-325-000540	1,00	1,00	1,00

hsa-miR-338-3p-002252	1,00	1,00	1,00
hsa-miR-340-002258	1,00	1,00	1,00
hsa-miR-369-5p-001021	1,00	1,00	1,00
hsa-miR-377-000566	1,00	1,00	1,00
hsa-miR-380-3p-000569	1,00	1,00	1,00
hsa-miR-409-5p-002331	1,00	1,00	1,00
hsa-miR-412-001023	1,00	1,00	1,00
hsa-miR-431-001979	1,00	1,00	1,00
hsa-miR-448-001029	1,00	1,00	1,00
hsa-miR-453-002318	1,00	1,00	1,00
hsa-miR-485-5p-001036	1,00	1,00	1,00
hsa-miR-487b-001285	1,00	1,00	1,00
hsa-miR-493-002364	1,00	1,00	1,00
hsa-miR-499-3p-002427	1,00	1,00	1,00
hsa-miR-504-002084	1,00	1,00	1,00
hsa-miR-506-001050	1,00	1,00	1,00
hsa-miR-508-5p-002092	1,00	1,00	1,00
hsa-miR-509-3-5p-002155	1,00	1,00	1,00
hsa-miR-510-002241	1,00	1,00	1,00
hsa-miR-515-5p-001112	1,00	1,00	1,00
hsa-miR-516a-5p-002416	1,00	5,80	0,17
hsa-miR-518a-3p-002397	1,00	1,00	1,00
hsa-miR-518a-5p-002396	1,00	1,00	1,00
hsa-miR-518c-002401	1,00	1,00	1,00
hsa-miR-520d-5p-002393	1,00	1,11	0,90
hsa-miR-521-001122	1,00	1,00	1,00
hsa-miR-524-5p-001982	1,00	1,00	1,00
hsa-miR-525-001174	1,00	1,00	1,00
hsa-miR-526b-002382	1,00	1,00	1,00
hsa-miR-541-002201	1,00	1,00	1,00
hsa-miR-544-002265	1,00	1,00	1,00
hsa-miR-556-3p-002345	1,00	1,00	1,00
hsa-miR-556-5p-002344	1,00	1,00	1,00
hsa-miR-651-001604	1,00	1,00	1,00
hsa-miR-653-002292	1,00	1,00	1,00
hsa-miR-654-001611	1,00	1,00	1,00
hsa-miR-654-3p-002239	1,00	1,00	1,00
hsa-miR-674-002021	1,00	1,00	1,00
hsa-miR-758-001990	1,00	1,00	1,00
hsa-miR-871-002354	1,00	1,00	1,00
hsa-miR-873-002356	1,00	1,00	1,00
hsa-miR-875-3p-002204	1,00	1,00	1,00
hsa-miR-885-3p-002372	1,00	1,00	1,00
hsa-miR-889-002202	1,00	1,00	1,00
hsa-miR-519e-002370	0,95	0,30	3,12
hsa-miR-561-001528	0,94	0,01	169,76
U6 snRNA-001973	0,92	0,23	4,01
hsa-miR-509-5p-002235	0,87	1,77	0,49
U6 snRNA-001973	0,79	0,28	2,86
U6 snRNA-001973	0,76	0,25	3,10
hsa-miR-520b-001116	0,69	0,01	70,83

hsa-miR-515-3p-002369	0,62	0,62	1,00
mmu-miR-124a-001182	0,62	0,10	6,46
hsa-miR-337-5p-002156	0,60	0,07	8,53
hsa-miR-886-3p-002194	0,54	2,16	0,25
hsa-miR-370-002275	0,53	0,09	5,79
hsa-miR-204-000508	0,49	2,45	0,20
hsa-miR-522-002413	0,48	0,10	5,01
hsa-miR-520a#-001168	0,46	0,46	1,00
hsa-miR-494-002365	0,42	0,26	1,58
hsa-miR-125a-5p-002198	0,41	0,05	8,71
mmu-miR-496-001953	0,35	0,35	1,00
hsa-miR-507-001051	0,33	0,33	1,00
hsa-miR-299-3p-001015	0,33	0,51	0,65
hsa-miR-487a-001279	0,28	0,04	7,51
hsa-miR-199b-000500	0,27	0,09	2,95
hsa-miR-208-000511	0,26	0,26	1,00
hsa-miR-485-3p-001277	0,25	0,60	0,42
hsa-miR-517c-001153	0,20	8,77	0,02
hsa-miR-299-5p-000600	0,20	0,02	9,64
hsa-miR-489-002358	0,17	0,01	17,16
hsa-miR-876-3p-002225	0,12	7,30	0,02
hsa-miR-433-001028	0,08	0,03	3,00
mmu-miR-379-001138	0,06	0,20	0,32
hsa-miR-381-000571	0,06	0,07	0,95
hsa-miR-518d-001159	0,05	0,01	4,73
hsa-miR-517b-001152	0,03	0,03	1,00
hsa-miR-375-000564	0,03	0,00	28,88
hsa-miR-129-000590	0,02	0,02	1,00
hsa-miR-888-002212	0,01	0,02	0,26
hsa-miR-218-000521	0,00	0,00	2,83
