# Supplementary material for: Oncogenic role of miR-155 in anaplastic large cell lymphoma lacking the t(2;5) translocation
Source: J Pathol. 2015 Apr 27;236(4):445–56. doi: 10.1002/path.4539 (PMC4557053; doi:10.1002/path.4539)
Supplement: Supplementary file 3 — Table S2. Regions analysed for CpG methylation close to the miR-155 host gene promoter: given are primer pairs of the analysed regions 14, 15, 16 and 18 [file path0236-0445-sd3.docx]

HERRINGTON EDITED

**Table S2.** Primers used for amplification of 4 regions close to the promoter of the miR-155 host gene

|  | **Forward** | **Reverse** |
| --- | --- | --- |
| miR-155.14 | AAGGTTTGGGTTTAATTTTTGTTT | TAAAAACACTCCTACCACCCAATTA |
| miR-155.15 | TTGTTTTTGGAATTTATAGTTTGAA | AAAACAAAAATTAAAACCCAAACCT |
| miR-155.16 | TGTAAAGAAGTTTAAGTATTAGTTT | TAACCTTAAAACCCCTACCTAATC |
| miR-155.18 | GTTGTAGGTTTTAAGAATAGGTAG | CTCCCTTCCCTAAACTACAAA |
