# Supplementary figures and images for: Oncogenic role of miR-155 in anaplastic large cell lymphoma lacking the t(2;5) translocation
Source: J Pathol. 2015 Apr 27;236(4):445–56. doi: 10.1002/path.4539 (PMC4557053; doi:10.1002/path.4539)

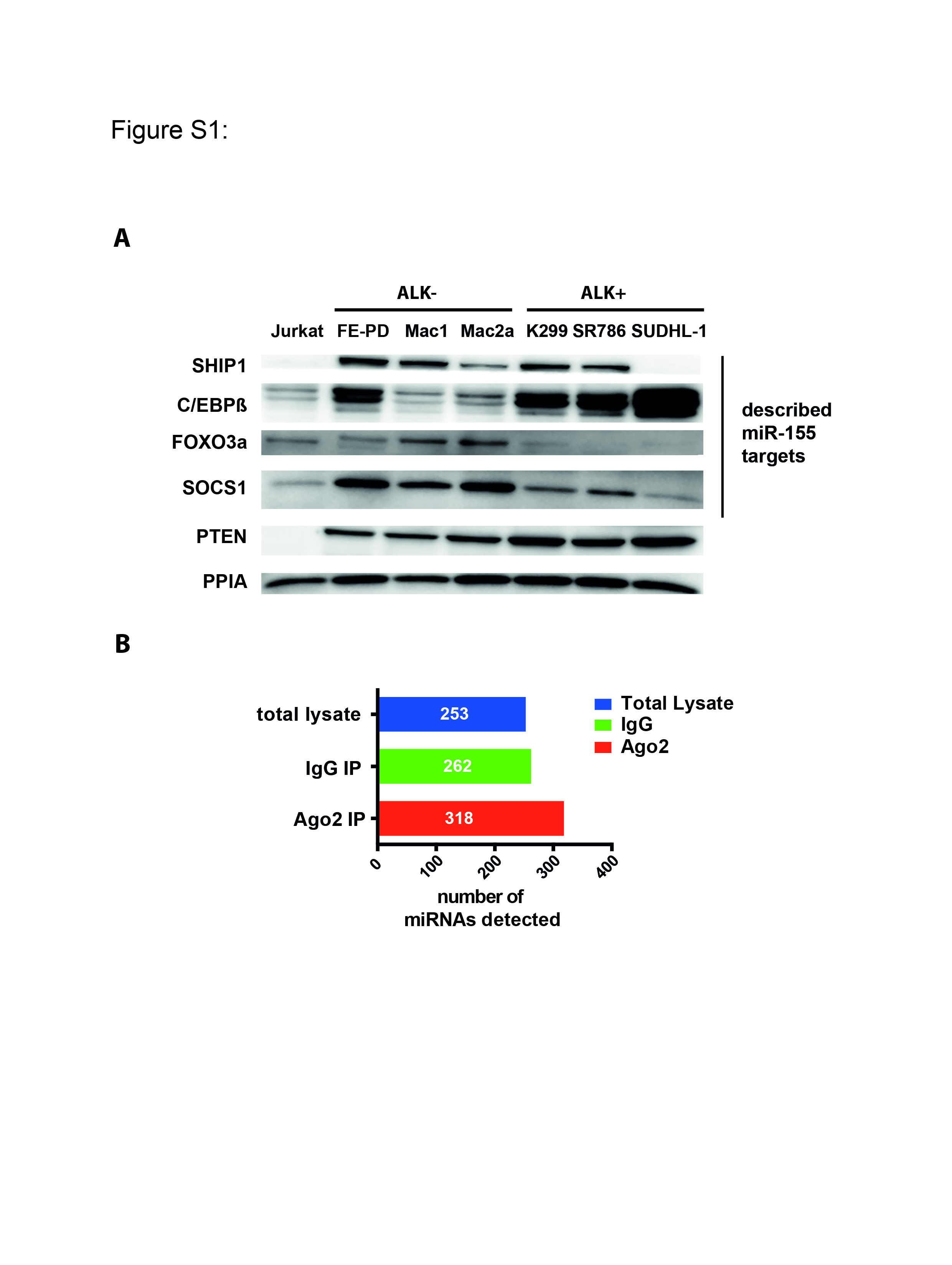

Supplement: Supplementary file 4 — Figure S1. Basal expression of miR-155 targets in ALCL cell lines and number of detectable miRNAs in the Ago2 IP fractions. (A) ALCL, ALK- (FE-PD, Mac1, Mac2a) and ALCL, ALK+ (Karpas-299, SR-786, SU-DHL-1) cell lines and the acute myeloid leukaemia cell line Jurkat were lysed, subjected to gel-electrophoresis and probed with antibodies against miR-155 targets SHIP1, C/EBPβ, FOXO3a and SOCS1. Since SHIP1 is a PIP3 lipid-phosphatase similar to PTEN, we also probed for PTEN. Levels of C/EBPβ were inversely correlated with miR-155 expression of cell lines, although the other described targets showed no correlation when basal levels were monitored. (B) After denaturation of proteins in the IP fractions, RNA was extracted and miRNAs were quantified using the Taq Man Array system v. 3 (Applied Biosystems). In the total lysate, IgG and Ago2 fraction 253, 262 and 318 miRNAs were detected, respectively [file path0236-0445-sd4.tif]

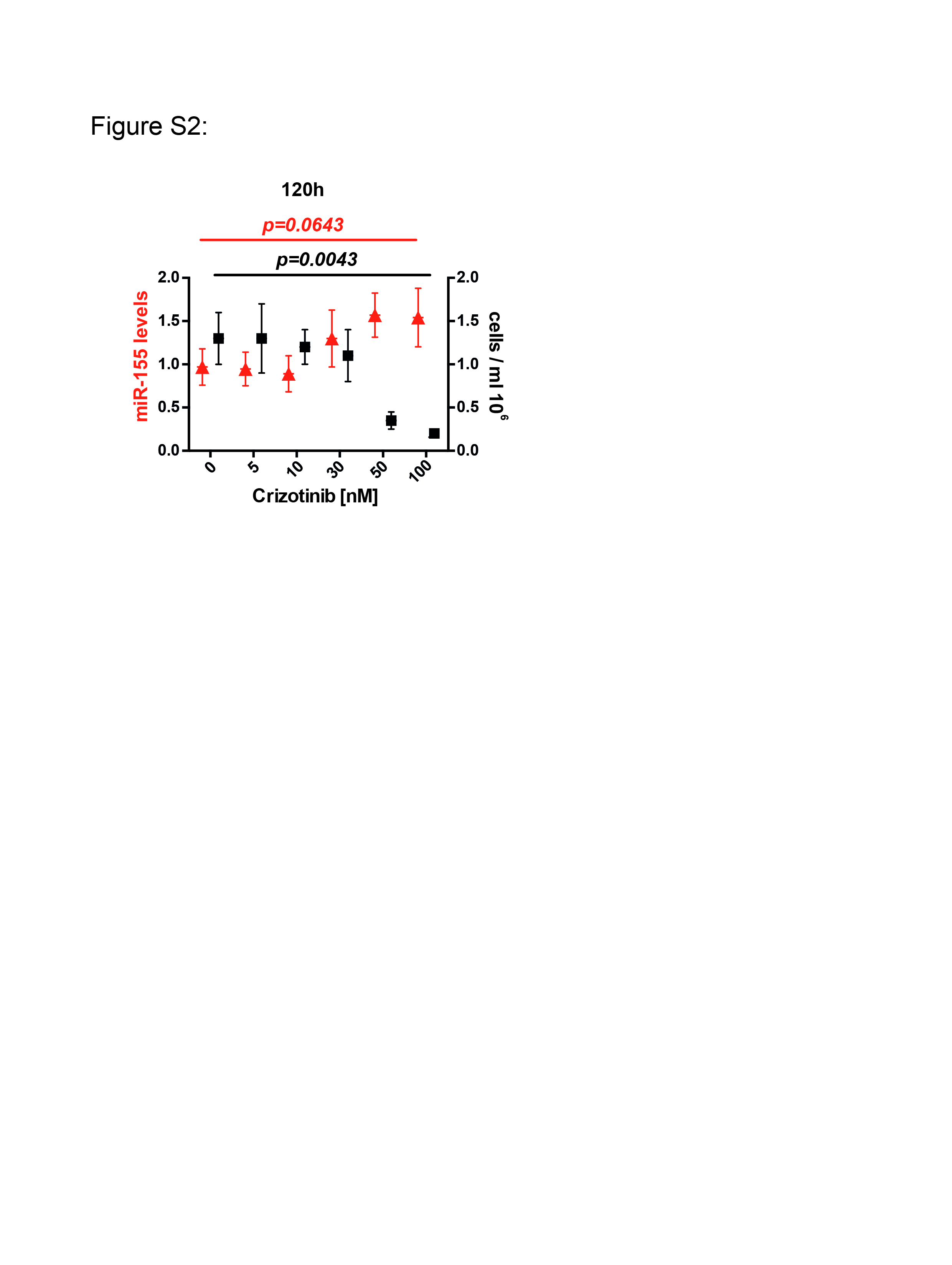

Supplement: Supplementary file 5 — Figure S2. miR-155 expression in response to ALK inhibition. The ALCL, ALK+ cell line Karpas-299 was treated for 120 h with 5, 10, 30, 50 and 100 nm crizotinib and cells were counted using the Moxi Z-Mini Automated Cell Counter from ORFLO technologies (Ketchum, ID, USA). In parallel, miR-155 levels were assessed by qRT–PCR. Despite the effective proliferation-reducing effect of crizotinib (p = 0.0043), only a small increase in miR-155 levels was observed, which was not significant (p = 0.0643); error bars represent mean ± SD [file path0236-0445-sd5.tif]

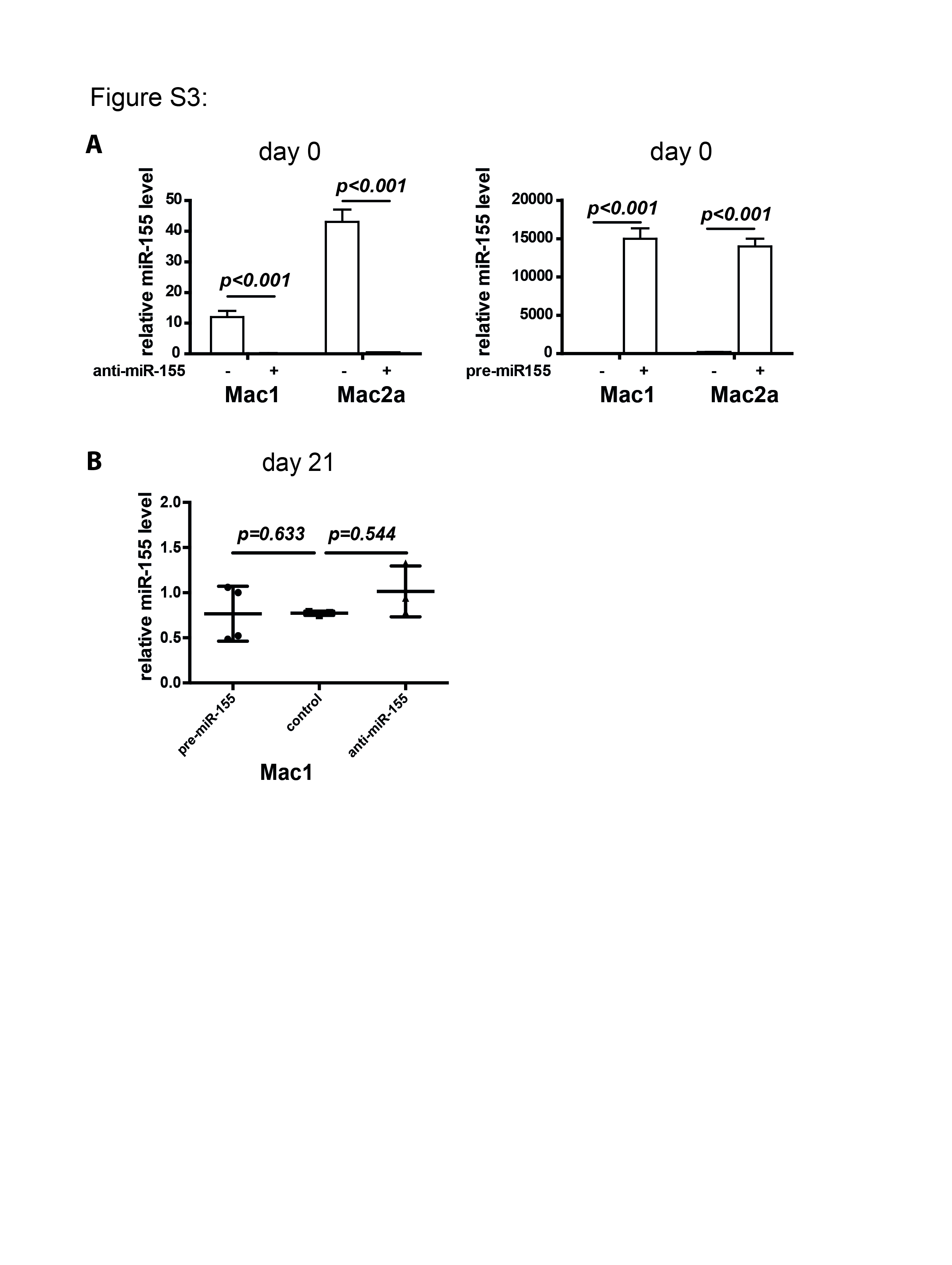

Supplement: Supplementary file 6 — Figure S3. miR-155 levels in cell lines used for the murine engraftment experiments. (A) The ALCL cell lines Mac1 and Mac2a were transfected with either anti-miR-155 mimics, pre-miR-155 mimics or non-targeting control RNA: 3 days after transfection, RNA was isolated, reverse-transcribed and probed for expression of miR-155 and miR-92 by qRT–PCR; on the same day, the cells were injected into mice, as described (day 0 of tumour growth). (B) At day 21, the mice were sacrificed and tumours prepared for immunostaining; RNA was isolated, reverse-transcribed and probed for expression of miR-155 and miR-92 by qRT–PCR [file path0236-0445-sd6.tif]

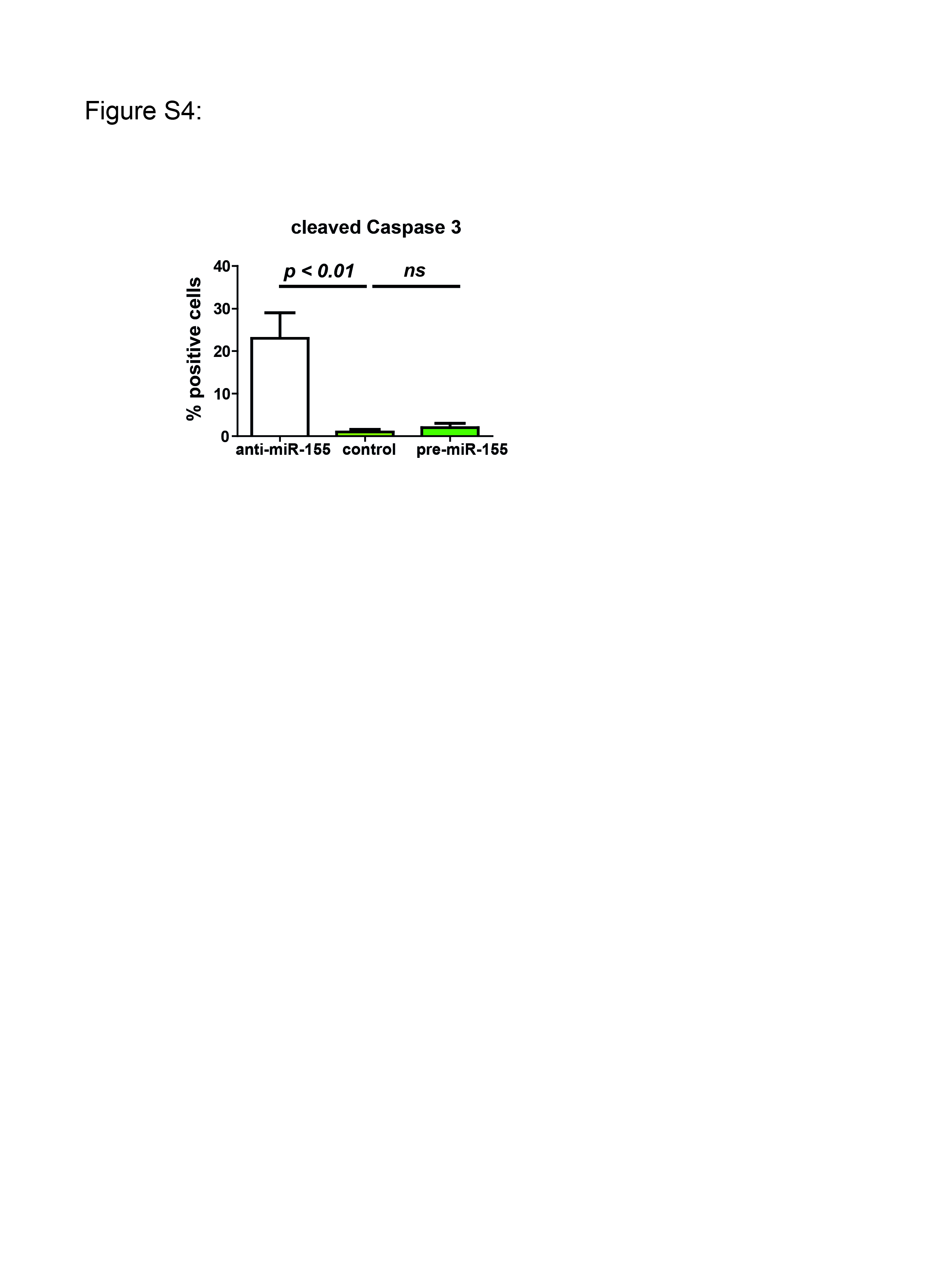

Supplement: Supplementary file 7 — Figure S4. Cleaved caspase 3 staining in murine tumours: cleaved caspase 3 IHC expression levels were quantified using HistoQuest software (TissueGnostics) [file path0236-0445-sd7.tif]

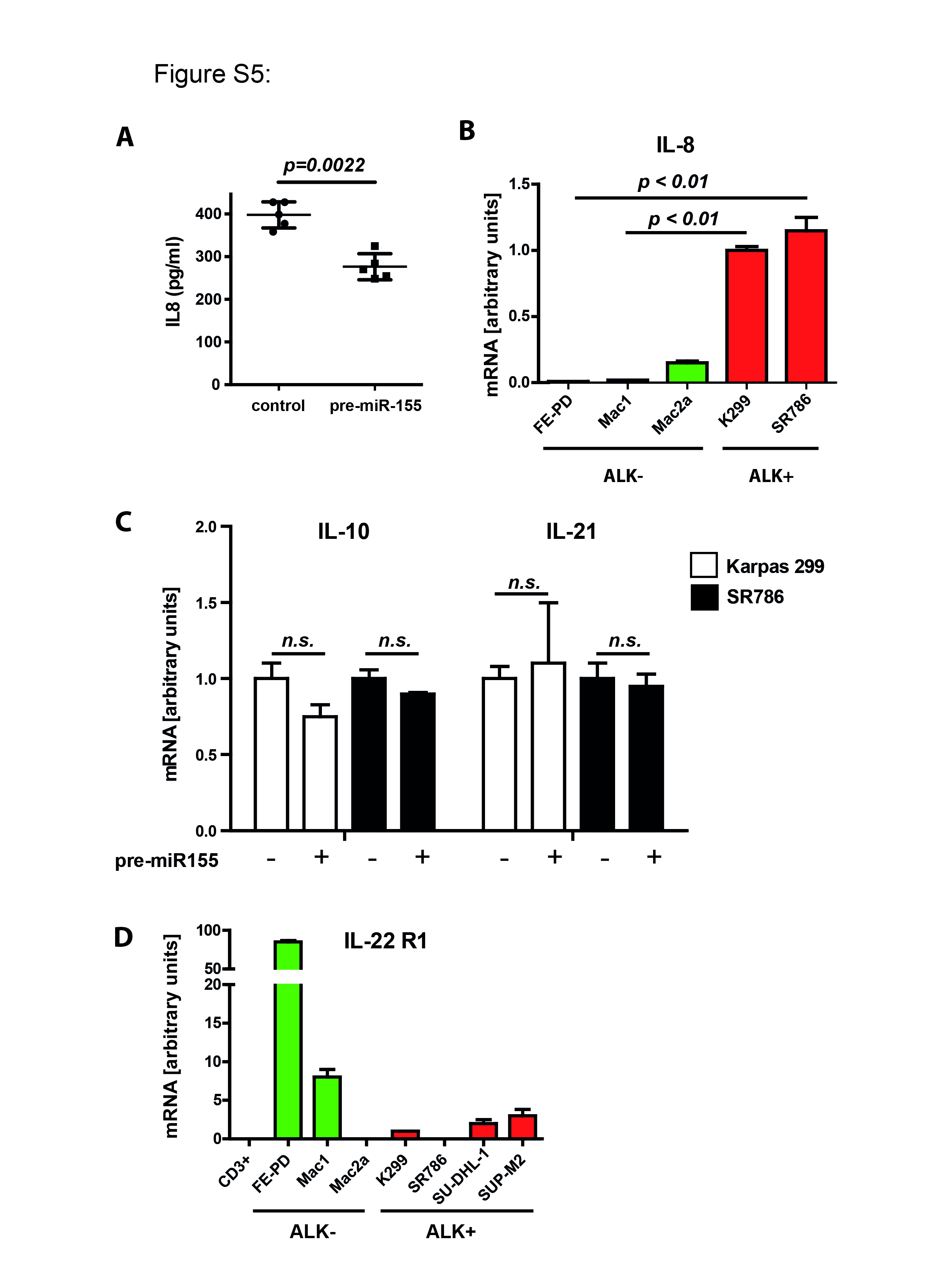

Supplement: Supplementary file 8 — Figure S5. miR-155 and cytokine expression in ALCL cell lines. (A) The ALCL cell lines Karpas-299 and SR786 were transfected with pre-miR-155 oligos or control oligos (Ambion): after 3 days, levels of IL-8 in the medium were quantified, using the IL-8 ELISA kit (e-bioscience); the experiment was carried out in five parallel chambers. For the SR786 cell line, IL-8 levels in the medium were below the detection limit. The result for the Karpas-299 cell line is shown. (B) RNA was isolated from different ALCL cell lines, reverse-transcribed and levels of IL-8 mRNA were determined by qRT–PCR. (C) ALCL, ALK+ cell lines Karpas 299 and SR786 were transfected with pre-miR155-mimics (+) or non-targeting control RNA (−): after 3 days, RNA was isolated, reverse-transcribed and IL-10 and IL-21 levels were measured by TaqMan qRT–PCR; neither cytokine was influenced by miR-155 transfection. (D) RNA was isolated from different ALCL cell lines, reverse-transcribed and levels of IL-22R determined by qRT–PCR; names of cell lines are given [file path0236-0445-sd8.tif]
